# Rational Drug Design of Axl Tyrosine Kinase Type I Inhibitors as Promising Candidates Against Cancer

**DOI:** 10.3389/fchem.2019.00920

**Published:** 2020-02-04

**Authors:** Edita Sarukhanyan, Sergey Shityakov, Thomas Dandekar

**Affiliations:** ^1^Department of Bioinformatics, Biocenter, University of Würzburg, Würzburg, Germany; ^2^Department of Anesthesia and Critical Care, University Hospital Würzburg, Würzburg, Germany; ^3^Department of Psychiatry and Mind-Body Interface Laboratory (MBI-Lab), China Medical University Hospital, Taichung, Taiwan; ^4^College of Medicine, China Medical University, Taichung, Taiwan

**Keywords:** Axl tyrosine kinase, anti-cancer drug-like molecules, *in silico* rational drug design, molecular docking, molecular dynamics

## Abstract

The high level of Axl tyrosine kinase expression in various cancer cell lines makes it an attractive target for the development of anti-cancer drugs. In this study, we carried out several sets of *in silico* screening for the ATP-competitive Axl kinase inhibitors based on different molecular docking protocols. The best drug-like candidates were identified, after parental structure modifications, by their highest affinity to the target protein. We found that our newly designed compound R5, a derivative of the R428 patented analog, is the most promising inhibitor of the Axl kinase according to the three molecular docking algorithms applied in the study. The molecular docking results are in agreement with the molecular dynamics simulations using the MM-PBSA/GBSA implicit solvation models, which confirm the high affinity of R5 toward the protein receptor. Additionally, the selectivity test against other kinases also reveals a high affinity of R5 toward ABL1 and Tyro3 kinases, emphasizing its promising potential for the treatment of malignant tumors.

## Introduction

Receptor tyrosine kinases are transmembrane proteins, which consist of several domains that are activated upon ligand binding to their extracellular regions, triggering downstream signaling cascades (Robinson et al., 2000; Myers et al., 2016). They are involved in various regulatory processes, such as cell survival, growth, differentiation, adhesion, proliferation, and motility (Robinson et al., 2000; Ségaliny et al., 2015; Myers et al., 2016). Impaired gene functions by mutations or deletions may cause the abnormal expression of protein kinases, which, in turn, entails tumor formation and progression (Blume-Jensen and Hunter, 2001; Zhang et al., 2008).

One of the frequently identified kinases involved in the formation of various types of tumors is Axl receptor tyrosine kinase (Craven et al., 1995; Sun et al., 2003). Axl belongs to the TAM family receptors, which also includes Tyro3 and Mer (O'Bryan et al., 1991; Li et al., 2009). The kinase structure comprises an extracellular part with two immunoglobulin (Ig)-like domains responsible for ligand binding, a transmembrane region, and an intracellular domain (O'Bryan et al., 1991; Lemke and Rothlin, 2008). The growth arrest-specific 6 (Gas6) protein precursor and protein S are primarily responsible for kinase activation as their ligands (Stitt et al., 1995; Varnum et al., 1995; Li et al., 2009). Both ligands share a similar domain composition. In particular, they include two sex-hormone-binding globulin domains at the C-terminus, both with the laminin G1 and G2 proteins necessary for the subsequent binding to the Ig-like domain of the receptor, causing their dimerization and activation (Lemke and Rothlin, 2008). Close to the N-terminal, there are epidermal-growth-factor-like repeats and, the so-called, Gla-domain that consists of gamma-carboxyglutamic acid, which is necessary for binding to phosphatidylserine of the apoptotic cell membrane in a vitamin-K-dependent reaction (Hasanbasic et al., 2005; Sasaki et al., 2006; Li et al., 2009).

Axl overexpression has been detected in a majority of human cancers, including acute myeloid leukemia (Rochlitz et al., 1999; Hong et al., 2008), breast cancer (Berclaz et al., 2001; Zhang et al., 2008; Gjerdrum et al., 2010), gastric (Wu et al., 2002) and lung cancer (Shieh et al., 2005), melanoma (Quong et al., 1994), osteosarcoma (Han et al., 2013), renal cell carcinoma (Gustafsson et al., 2009), etc. Therefore, targeting the Axl to inhibit its function might be a promising strategy for the treatment of various malignant tumors. Different strategies of targeting the Axl have already been considered. For instance, Rankin and Giaccia (2016), in their review, highlight the three classes of Axl inhibitors directed on cancer therapy. The first class includes small-molecule tyrosine kinase inhibitors that block Axl kinase activity (Rankin and Giaccia, 2016). The second class consists of anti-Axl antibodies (Rankin and Giaccia, 2016) that block Axl activation, which is triggered by the Axl–Gas6 interaction, and the third class comprises soluble Axl decoy receptors (Rankin and Giaccia, 2016) that serve as a trap for Gas6, hence, preventing the Axl–Gas6 binding.

Different experimental and computational techniques have been developed and applied in the last decades for rational drug design and discovery (Baldi, 2010; Ou-Yang et al., 2012; March-Vila et al., 2017). For instance, computational and experimental approaches focused on *in silico* design and organic synthesis of the Axl kinase inhibitors have already been performed by Mollard et al. (2011). In their research, the authors constructed a homology model for the active site of the Axl kinase and performed docking experiments for the designed compounds. Recently, the three-dimensional (3D) structure of the Axl kinase in a complex with its inhibitor (macrocyclic compound 1) has been successfully solved by Gajiwala et al. (2017) using differential scanning fluorimetry and hydrogen–deuterium exchange mass spectrometry. This 3D structure, as a tetrameric configuration, consists of two active (B and D chains) and two inactive (A and C) motifs in a complex with a small ATP-competitive inhibitor. The active and the inactive states are characterized by the DFG (Asp-Phe-Gly) loop-in and loop-out conformations.

According to the mode of binding, all tyrosine kinase inhibitors have been divided into different types. In their review, Zhang et al. (2009) distinguishes four basic types of inhibitors. According to this classification, the type I and the type II inhibitors bind to the DFG-in and DFG-out motifs, respectively. Additionally, the type III inhibitors interact with the protein outside the highly conserved ATP-binding pocket, representing allosteric binding and, therefore, named as allosteric inhibitors (Wu et al., 2015). Finally, the type IV inhibitors bind the active site irreversibly, forming covalent bonds within the binding pocket. A slightly expanded classification of the protein kinase inhibitors is suggested by Roskoski (2016).

In the current study, we introduce systematic computational analysis for the DFG-in conformation of the Axl kinase to inhibit its activity using different sets of molecular docking algorithms and molecular dynamics simulation techniques to handle cancer-related diseases.

## Computational Methods

### 3D Structure Derivation

The 3D coordinates for the Axl kinase in a complex with the macrocyclic compound 1 have been retrieved from protein data bank (PDB) under the reference code 5U6B. Other kinases such as ABL1, ALK5, FYN, JAK, MER, MET, and Tyro3 have been derived under the 4WA9, 3GXL, 2DQ7, 5WO4, 5U6C, 2WD1, and 3QUP PDB codes, respectively. Coordinates for the Axl kinase type I inhibitors have been obtained from the PubChem under the following CIDs: 46215462 (R428), 11282283 (Amuvatinib), 5328940 (Bosutinib), 11626560 (Crizotinib), 49803313 (Gilteritinib), 49870909 (S49076), 46870258 (SGI-7079), 5329102 (Sunitinib), 56839178 (TP-0903), and 73425588 (UNC2025). The PubChem CIDs for the 136 R428 patented analogs as well as the 26 analogs of crizotinib can be found in the Supplementary Material.

### Structure Preparation

All compounds were energy-minimized prior to docking, with the help of the Molecular Operating Environment (MOE) software [Molecular Operating, Environment (MOE), 2016], using MMFF94 (Merck Molecular Force Field) with the gradient convergence set to 0.01 kcal/mol and saved in.pdb and.mol2 formats. Further structural modifications of the best-scoring compounds were also performed with the help of the MOE software [Molecular Operating, Environment (MOE), 2016]. Protein structure refinement as well as ligand libraries have been prepared with the tools of the same software.

### Docking

Molecular docking simulations have been performed with the help of well-validated (Chan and Labute, 2010; Forli et al., 2016) software such as GOLD (Jones et al., 1997), MOE [Molecular Operating, Environment (MOE), 2016], and AutoDock (Morris et al., 2009).

#### GOLD

Molecular docking using the GOLD software was performed using version 5.5 (Jones et al., 1997). The binding site residues were defined by specifying the crystal structure ligand coordinates bound to the protein and using the default cutoff radius of 6 Å, with the “detect cavity” option enabled. The GOLD docking experiments were performed using the ChemPLP scoring function. For each compound, 50 complexes were generated. The highest-scoring compounds were selected as the most appropriate ones.

#### MOE

Docking has been performed by selecting the default “Rigid Receptor” protocol. As a binding site, the coordinates of co-crystalized ligand atoms have been selected. The ligand placement was performed using the Triangle Matcher protocol. The top 30 poses were ranked by London dG scoring function, and the resulting five poses were identified using the generalized-Born-volume-integral/weighted-surface-area function. The conformations with the more negative final score were considered as favorable.

#### AutoDock

Molecular docking using AutoDock software (Morris et al., 2009) has been performed using version 4.2.6 (available at: http://autodock.scripps.edu). The AutoDock tools were used to generate the input parameter files for docking. For the current study, the receptor was considered as a rigid molecule, while the ligands contained rotatable bonds. Pure protein was applied for the docking, while all non-protein moieties were removed. Additional hydrogen atoms were added to the receptor, and the new PDB coordinates were saved. The ligand PDB file was modified by the addition of groups representing the Gasteiger charges. The volume of the grid box was set as 50 × 50 × 50 Å, with 0.375-Å spacing. The center of the grid box was placed so that it coincided with the center of the co-crystalized structure of the compound (macrocyclic compound 1). A genetic algorithm was selected to set the search parameters. The number of docking runs was fixed to 50. The conformations with the lowest binding energies have been selected for further analysis.

The figures were prepared using the UCSF Chimera (Pettersen et al., 2004; available at: http://www.rbvi.ucsf.edu/chimera) and the MOE [Molecular Operating, Environment (MOE), 2016] software.

### Molecular Dynamics

All molecular dynamics (MD) simulations were performed using the AMBER 16 package (Case et al., 2005) with the FF99SB and GAFF force fields for the Axl protein and its ligands. The systems were solvated with the TIP3P water models and neutralized by adding the Na^+^ ions using the tLEap input script available from the AmberTools package. Long-range electrostatic interactions were modeled via the particle–mesh Ewald method (Essmann et al., 1995). The SHAKE algorithm (Miyamoto and Kollman, 1992) was applied to constrain the length of covalent bonds, including the hydrogen atoms. Langevin thermostat was implemented to equilibrate the temperature of the system at 300 K. A 2.0-fs time step was used in all of the MD setups. For the minimization and equilibration (NVT ensemble) phases, 10,000 steps and 1-ns time period were used, respectively. Finally, 50-ns classical MD simulations, with no constrains as NPT ensemble, were performed for each of the protein–ligand complexes using the molecular mechanics combined with the Poisson–Boltzmann (MM-PBSA) or generalized Born (MM-GBSA) augmented with the hydrophobic solvent-accessible surface area term (Kollman et al., 2000; Shityakov et al., 2017). The MM-PBSA/GBSA solvation models were applied as a post-processing end-state method to calculate the free energies of molecules in the solution by means of the optimized python script (MM-PBSA.py).

## Results

### Validation of the Binding Poses

**Figure 1:**
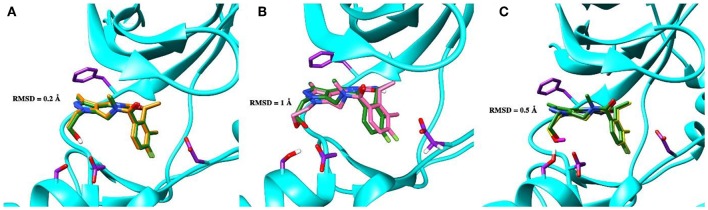
The superposition of the crystal structure of macrocyclic compound 1 (shown in *forest green*) with its docked pose performed by the GOLD (**A**, *orange*), MOE (**B**, *deep pink*), and AutoDock (**C**, *yellow*) software. The protein is represented as a secondary structure and highlighted in *cyan*. Some of the pocket residues are shown in licorice and colored in *purple*.

To validate the poses of the ligands, we performed docking of the co-crystalized ligand–macrocyclic compound 1 (Gajiwala et al., 2017) to the ATP-binding pocket of the Axl kinase using the GOLD, MOE and AutoDock simulation software. Figure 1 demonstrates the superposition of the crystal structure of the ligand (always shown in forest green) and the docked pose of the same ligand performed by the GOLD (orange, Figure 1A), MOE (deep pink, Figure 1B), and AutoDock (yellow, Figure 1C) software. The calculated RMSD values for this ligand are below the commonly accepted threshold of 2.0 Å, indicating the validity of the above-mentioned docking engines for the prediction of the ligand binding pose. The most accurate results were reproduced by the GOLD and AutoDock software, with the calculated RMSD values of 0.2 and 0.5 Å, respectively (Figures 1A,C). A less accurate result was shown by the MOE software, where the RMSD between the crystal structure and the docked pose was 1 Å (Figure 1B).

### Docking of ATP-Competitive Type I Inhibitors

The first round of *in silico* testing was performed on the commercially available ATP-competitive Axl kinase type I inhibitors using the molecular docking protocols. From Table 1, it is clear that the scoring functions from the molecular docking results indicate a better affinity for the analyzed drugs in comparison to the ATP, which was used here as a reference. The top scores belong to macrocyclic compound 1, crizotinib, and R428 according to the three independent molecular docking protocols. The last two substances were chosen as the core components for the structure similarity search and *in silico* chemical modifications based on their ability to block specific kinases that are highly expressed in malignant cells (Solomon et al., 2014; Chen et al., 2018).

**Table 1:** Scores for the ATP and the ATP-competitive type I kinase inhibitors obtained from the docking performed by the GOLD, MOE, and AutoDock software.

**Compound**	**Score (GOLD)**	**Score, kcal/mol (MOE)**	****Δ***G*, kcal/mol (AutoDock)**
ATP	55.59	−7.34	−4.43
Bosutinib	60.44	−7.60	−8.94
Gilteritinib	61.44	−8.29	−9.15
SGI-7079	61.96	−7.57	−8.80
TP-0903	66.83	−7.58	−8.74
*Crizotinib*	*79.32*	*−7.78*	*−9.11*
Amuvatinib	63.15	−7.16	−8.37
UNC2025	72.72	−7.26	−9.64
S49076	66.97	−7.04	8.08
Sunitinib	58.35	−6.8	−7.25
Compound13	69.5	−6.97	−9.62
*Macrocyclic compound 1*	*78.98*	*−7.64*	*−9.14*
*R428*	*75.89*	*−7.59*	*−10.04*

*The top three scores are highlighted in italics*.

### Molecular Docking of R428-Based Analogs

**Figure 2:**
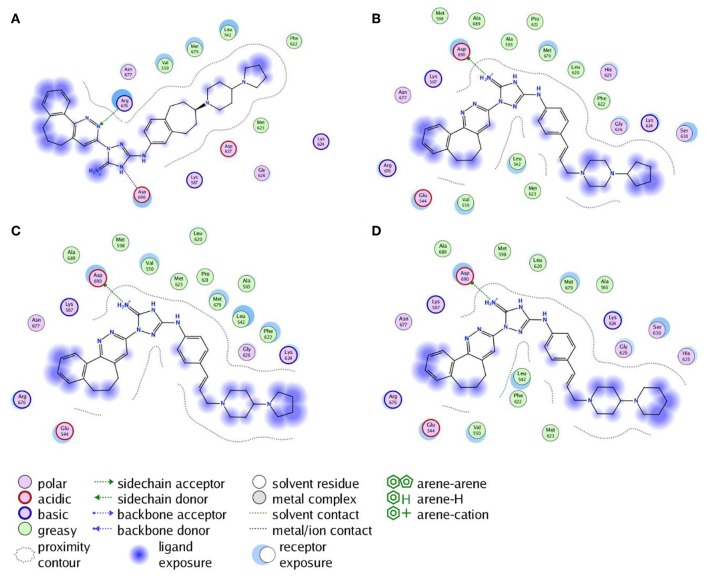
The 2D interaction diagrams of the R428_1 **(A)**, R428_2 **(B)**, R428_3 **(C)**, and R428_4 **(D)** compounds with the Axl kinase pocket.

**Figure 3:**
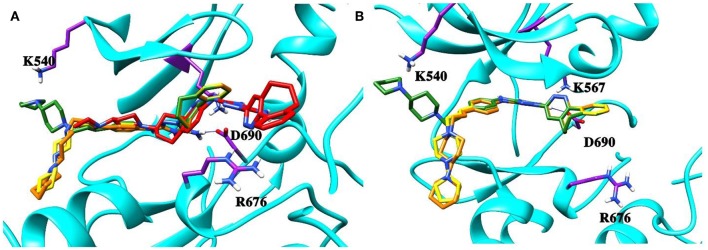
**(A)** The superposition of the top four out of best 10 R428 analogs according to the MOE and GOLD docking results. The compound R428_1 is shown in *red*, the compound R428_2 is shown in *orange*, the compound R428_3 is shown in *yellow*, and the compound R428_4 is shown in *green*. The backbone of the kinase is represented as a cartoon and shown in *cyan*; the key residues inside the pocket are highlighted in *purple*. The hydrogen bond between the R428_2, R428_3, and R428_4 compounds and D690 is marked as a *blue line*. **(B)** Superposition of the R428_2, R428_3, and R428_4 compounds for a better representation of their conformational coincidence.

Next, the drug R428 was subjected to the structure similarity search using the PubChem search algorithm (Kim et al., 2016). The results came up with 136 patented analogs, which are suitable for the GOLD/MOE molecular docking to determine the top 10 hit molecules (Table 2). Within this group the best four compounds (Table 3) with PubChem CID numbers 67104315 (R428_1), 67104254 (R428_2), 67103757 (R428_3), and 67103760 (R428_4) were selected for further analysis. The 2D protein–ligand interaction diagrams (see Figures 2A–D) were drawn for these molecules to demonstrate the binding modes of the ligands within the Axl receptor binding pocket. So, the amino groups of R428_2-4 establish the interaction with D690. Besides an interaction with D690, an additional interaction was observed between the aromatic ring of compound R428_1 and N676. Moreover, the R428_2-4 ligands adopt similar conformational rearrangements within the Axl binding pocket as observed from Figures 3A,B. The calculated RMSD values for the R428_4 and R428_2 compounds, with respect to the reference R428_3 compound, are 0.8 and 2.3 Å, respectively. On the other hand, R428_1 is more shifted with respect to the plane of the other three compounds (see Figure 3A) due to the bigger number of rings in the molecule, thus reducing its flexibility. The RMSD value in this case is 5.7 Å.

**Table 2:** The PubChem CID and scores for the top 10 out of 136 R428 analogs according to the docking results performed by the GOLD and MOE software.

**No**.	**GOLD**		**MOE**	
	**PubChem CID**	**Score**	**PubChem CID**	**Score, kcal/mol**
1	*67104315*	88.1	25127087	−8.7
2	46843782	83.3	*67103760*	−8.6
3	90974101	83.5	46843985	−8.6
4	*67104254*	83.6	*67103757*	−8.5
5	*67103757*	84.1	*67104254*	−8.5
6	46843846	82.3	66694833	−8.4
7	67537596	81.1	25126441	−8.4
8	*67103760*	82.6	67103984	−8.4
9	46843917	81.2	*67104315*	−8.4
10	46843983	80.7	67104240	−8.2

*The CIDs of the compounds corresponding to the best scores are shown in italics*.

**Table 3:** Four best R428 analogs according to the GOLD and MOE molecular docking scores.

**Compound**	**PubChem CID**	**2D structure**	**Score (GOLD)**	**Score, kcal/mol (MOE)**
R428_1	67104315	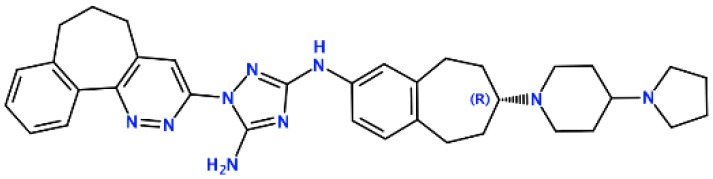	88.1	−8.6
R428_2	67104254	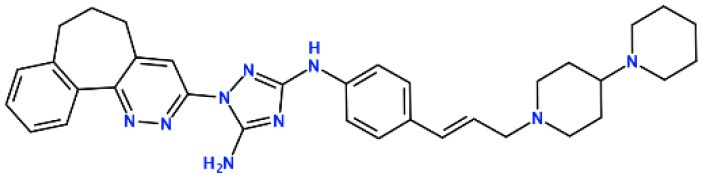	83.6	−8.5
R428_3	67103757	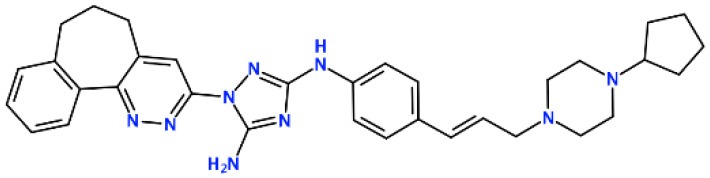	84.1	−8.5
R428_4	67103760	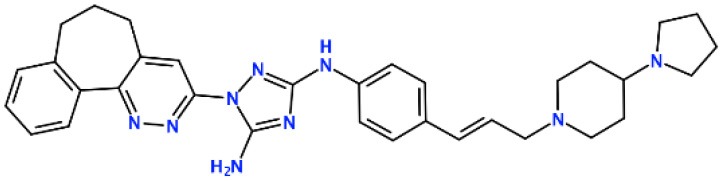	82.6	−8.6

### Molecular Docking of Crizotinib-Based Analogs

A similar structural search was applied to crizotinib to ensure the identification of the crizotinib-based analogs. The search results provided 26 analogs for further GOLD/MOE screening. Finally, the top five compounds were determined (Table 4), but none of them was subjected to *in silico* chemical modifications due to their lesser binding affinity than crizotinib itself. Therefore, the parental structure was modified to improve its binding properties toward the Axl kinase.

**Table 4:** The PubChem CID and the scores for the top five out of 26 crizotinib derivatives according to the docking results performed by the GOLD and MOE software.

**No**.	**GOLD**		**MOE**	
	**PubChem CID**	**Score**	**PubChem CID**	**Score, kcal/mol**
1	56671943	80.4	11656580	−7.9
2	54579455	80.2	54579455	−7.7
3	11647760	77.5	56671943	−7.6
4	72199381	77.1	11662380	−7.4
5.	11612136	76.0	11576617	−7.4

### *In silico* Design of the Refined Compounds

#### R428_1 Modifications

**Figure 4:**
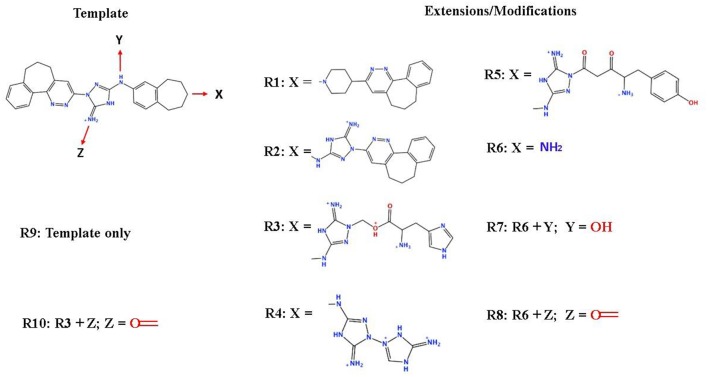
The design strategy of the new compounds starting from the R428_1 compound (see Table 3). The upper-left part is an invariant part of the compound used as a template. The upper/lower right and lower left parts are extensions/modifications of the template.

To enhance the binding affinity of the identified top compounds (Table 3), we devised the structural modification scheme for R428_1. Its invariant part was estimated from the best protein–ligand binding mode, forming the “scaffold” to adjust the ligand conformation inside the binding pocket (Figure 4). Therefore, we decided to design the new compounds by implementing this part as a template and adding the molecular extensions (X, Y, and Z) into the “scaffold” at the locations indicated by the arrows. So, the first six compounds (R1–R6) were designed: the compound R1 has been derived by adding piperidine bound to a tri-cyclic moiety (see Figure 4), the compound R2 was formed by adding a triazole-like ring also connected with a tri-cyclic moiety, the compound R3 was derived by adding a histidine-bound triazole ring, the compound R4 was formed by adding two repeats of triazole-like rings, the compound R5 was achieved by extending the template structure with a tyrosine-bound triazole-like ring, and the compound R6 has been derived by adding to the template an NH_2_ group. The next four compounds (R7–R10) have been derived from the above-mentioned designed R6 and R3 compounds with a slight alteration of the template structure. Thus, the compound R7 was obtained by replacing a hydrogen atom with a hydroxyl group in the compound R6. The compound R8, in turn, was obtained by replacing an amino group with a nitroso group in the compound R6. The compound R9 represents a pure template (see the upper-left part of Figure 4). Finally, the compound R10 has been derived by replacing an aromatic amino group with a nitroso group in the compound R3.

**Figure 5:**
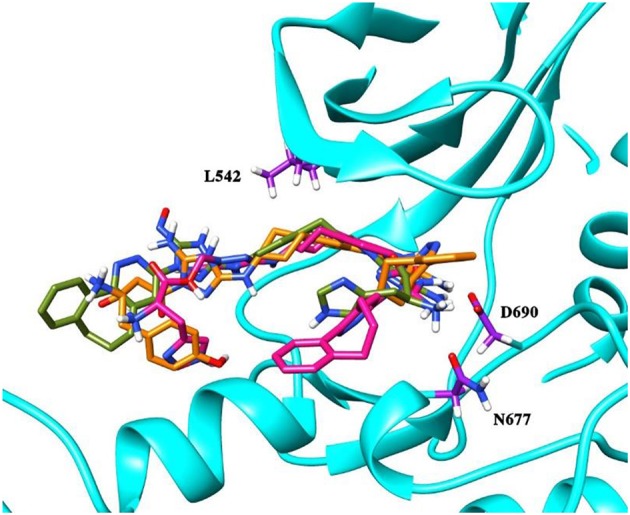
The superposition of the three top-scoring compounds designed. The compound R3 is shown in *deep pink*, the compound R5 is shown in *orange*, and the compound R10 is shown in *olive green*. The backbone of the kinase is represented as a secondary structure and highlighted in cyan; some of the key residues in the binding pocket—D690, N677, and L542—are shown as licorice and indicated in *purple*.

The newly designed compounds were tested by docking to the ATP-binding site of the Axl kinase. Improved binding results according to the GOLD, MOE, and AutoDock software were obtained for the designed R3, R5, and R10 compounds (see Table 5). In particular, they score higher than the top R428 derivatives (see Table 2). The superposition of R3, R5, and R10 inside the ATP-binding pocket of the Axl kinase shows a similar level of spatial occupancy (see Figure 5). However, shifts as well as differences in orientations of the tri-cyclic rings are observable. The compound R3 (Figure 5, deep pink) is oriented so that it obtains a “horseshoe-like” conformation inside the binding pocket and points its tri-cyclic moiety toward N677, the compound R5 (Figure 5, orange) obtains a slightly extended conformation and exposes its tri-cyclic ring to D690, while the compound R10 (Figure 5, olive green) is oriented opposite from the R3 and the R5 direction, pointing an amino group toward D690.

**Table 5:** Docking scores according to the GOLD, MOE, and AutoDock software for the newly designed compounds—modifications of R428_1.

**Compound no**.	**2D structure**	**Score (GOLD)**	**Score, kcal/mol (MOE)**	**Δ*G*, kcal/mol (AutoDock)**
R1	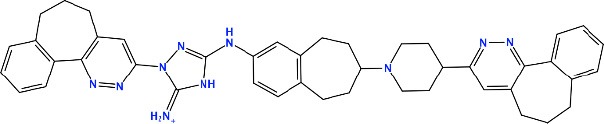	74.0	*−9.4*	−6.4
R2	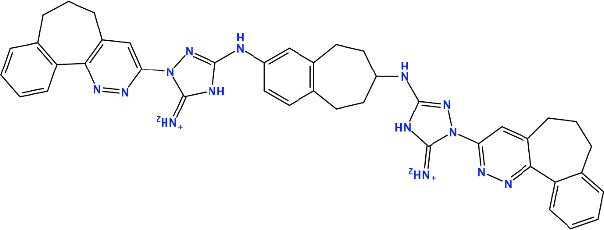	*89.6*	−9.2	*−11.0*
*R3*	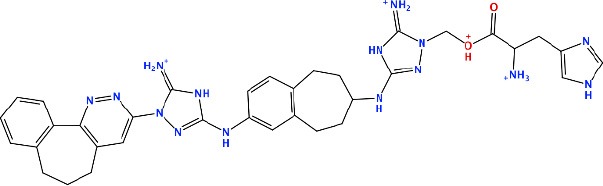	*91.3*	*−10.5*	*−10.7*
R4	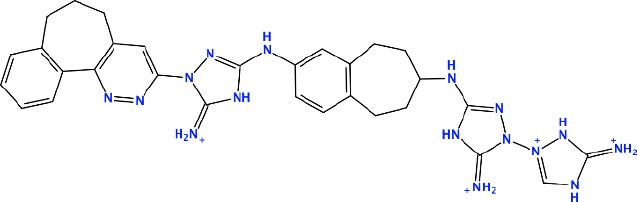	80.5	−8.0	−10.5
*R5*	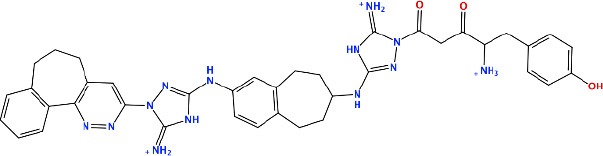	*98.6*	*−10.0*	*−11.7*
R6	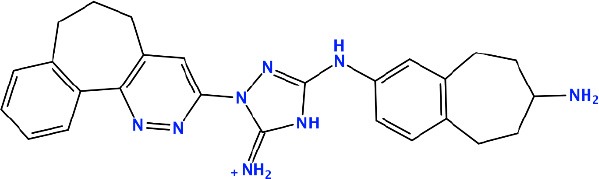	66.7	−7.2	−10.7
R7	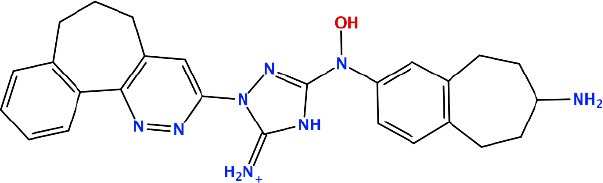	69.2	−7.3	−10.7
R8	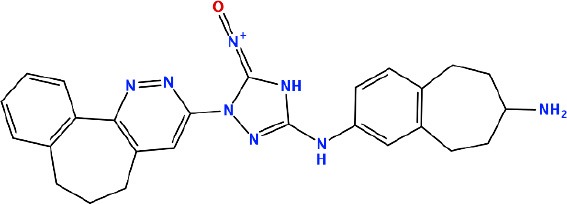	70.8	−8.0	−10.6
R9	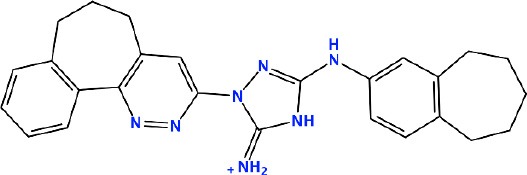	69.7	−7.3	−10.4
*R10*	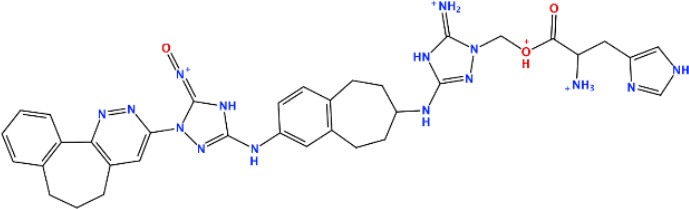	*93.7*	*−10.0*	*−11.1*

*The top four scores are highlighted in italics*.

All of the newly designed compounds, except R4 and R8, form hydrogen bonds (H-bonds) with D690 (see Table 6). The compounds R1–R3 and R10 form an H-bond with N677, while the compounds R2–R4, R6, and R8 form an H-bond with M623. Additionally, the compound R2 forms an H-bond with G626, the compound R3 forms an H-bond with K624, the compound R4 forms an H-bond with A562, the compound R5 forms an H-bond with H625, and the compound R8 forms an H-bond with P621. Van der Waals interactions have been observed as well between the pocket residues and the designed compounds. So, the compounds R1–R5, R7, R9, and R10, besides the above-mentioned residues, additionally make interactions with L542. The compounds R6 and R8, in turn, make interactions with A565.

**Table 6:** The list of cavity residues involved in the H-bond formations as well as in the short-range van der Waals interactions with the designed compounds R1–R10 according to the GOLD software.

**Compound no**.	**H-bond**	**van der Waals interactions**
R1	D690, N677	H629, D690, G626, R676, M629, F622, V550, L542
R2	D690, N677, M623, G626	D690, H629, A565, L542, M623
R3	D690, N677, M623, K624	D690, R676, L542, M623, K624, H625
R4	A562, M623	D690, R676, M623, K624, L542, A565
R5	D690, H625	D690, M623, L542, G626
R6	D690, M623	A565, M623, R676
R7	D690	L542, K567, E546, G545
R8	D627, M623, P621	M598, V550, A565, P621, M623
R9	D690	M598, L542, D690, K567, E546, G545
R10	K624, D627, N677, D690	D639, K624, L542, N677

#### R428_2, R428_3, and R428_4 Modifications

**Figure 6:**
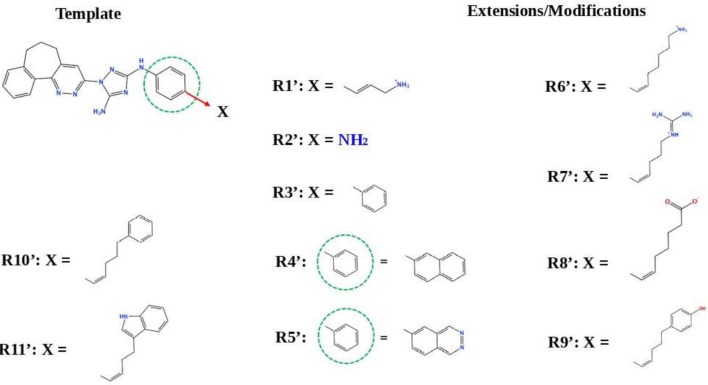
The strategy of the design of the new compounds based on the R428_2, R428_3, and R428_4 template extension. The upper-left part is a template. The upper- and lower-right parts are extensions/modifications of the template.

Next, we tried to obtain new compounds with improved binding based on the R428_2, R428_3, and R428_4 structural characteristics. Since these compounds share an almost identical structure and acquire a quite similar conformation inside the pocket (see Figures 2, 3B), we selected a common structural feature that is shared among these compounds (see the upper-left part in Figure 6) and used it as a template for further chemical modifications. Figure 6 describes a stepwise process of the design of the new (R1′-R11′) compounds. So, the compound R1′ has been derived by the addition of but-2-enylazanium to the part indicated with an arrow (upper-left part of Figure 6). The compound R2′ has been derived by adding an amino group, the compound R3′ has been formed by adding an aromatic ring, the compound R4′ has been derived by replacing a single aromatic ring (indicated with a dashed green circle) in a template by double aromatic rings, and the compound R5′ has been formed by the replacement of two aromatic carbons with double-bonded nitrogen atoms in the structure of R4′. The compound R6′ has been obtained by adding an extended aliphatic chain to the template, and the compound R7′ has been derived by adding a chain with an arginine-like termination. The compound R8′ has been derived similarly to compound R7′, however with a carbonyl termination. The compound R9′ has been formed by adding to the aliphatic extension an aromatic ring containing a hydroxyl group, the compound R10′ has been obtained similarly to the compound R9′, however with the absence of a hydroxyl group, and, finally, the compound R11′ contains an extension with a tryptophan-like termination.

Table S1 reports the docking scores for the above-mentioned modifications. The compounds did not show significant improvements of the binding scores compared to the original compounds—R428_2, R428_3, and R428_4 (please refer to Table 2 and Table S1). However, improvements were noticed for the designed compounds R9′ and R10′. For both compounds, the scores were ~85 (by GOLD) and −8 (by MOE) (see Table S1). We, therefore, did not perform a further analysis on these compounds as they did not demonstrate binding affinities that are stronger than the ones of R428_1 modifications (please refer to Table 5).

#### Crizotinib Modifications

**Figure 7:**
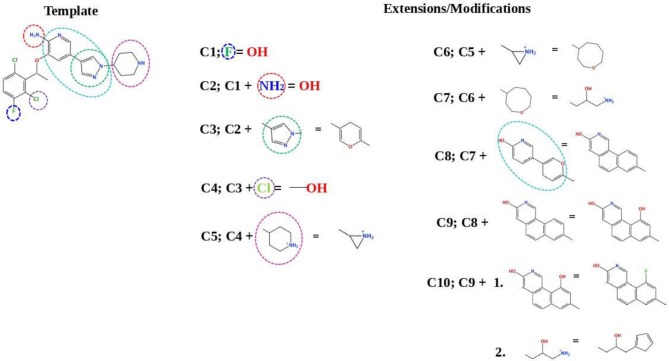
A schematic representation of the crizotinib modifications. The upper-left part is a template that undergoes modifications (indicated by *dashed circles*). The upper-right and lower-right/left parts are extensions/modifications of the template.

Figure 7 describes the crizotinib modifications. In the upper-left part of the figure, the 2D structure of crizotinib is demonstrated. The strategy of the design is based on the following scheme: each consecutive compound is derived from a previous one via the modification of a single group present in the structure. The parts that underwent modifications are indicated with dashed circles. Thus, the compound C1 is derived by the replacement of a fluorine atom, indicated with a violet dashed circle, with a hydroxyl group; the compound C2 is derived from compound C1 by the replacement of an amino group, indicated with a red dashed circle, with a hydroxyl group; the compound C3 is derived from compound C2, where the pyrazole ring, indicated with a dashed green circle, is replaced by a pyran ring; the compound C4 is derived from the compound C3 by the replacement of a chlorine atom, indicated by a purple ring, with a hydroxyl group; the compound C5 is derived from the compound C4 by the replacement of an aminocyclohexane group, indicated with a dashed magenta circle, with a cyclopropane group; the compound C6 is derived from the compound C5 by the replacement of a cyclopropane group with cyclooctane; the compound C7 is derived from the compound C6 by the replacement of cyclooctane with 3-hydroxy-5-amino-pentane, the compound C8 is derived from the compound C7 by the replacement of two cyclohexanes, indicated with a dashed cyan circle, with a triple aromatic network; the compound C9 is derived from the compound C8 by adding to a triple aromatic network one more hydroxyl group, and, finally, the compound C10 is derived from the compound C9 by double modifications: first, by the replacement of a hydroxyl group in a triple aromatic network with fluorine atoms and, second, by the replacement of 3-hydroxy-5-amino-pentane with 1-cyclopenta-1,3-dien-1-ylbutan-2-ol.

Table S2 shows the docking results for the crizotinib modifications—compounds C1–C10. The possible alterations in the structure of crizotinib did not result in a significant binding improvement. Therefore, we did not consider these new derivatives for further analysis.

### Validation of Docking Results

To estimate the strength of our best-designed compound R5, we have compared the calculated inhibition constants (*K*_i_) as well as the ligand efficiencies (LE) for ATP, R428, R428_1, and R5. The data are reported in Table 7. As the results show, the highest value of inhibition constant belong to ATP (566 μM). R428 shows the value of *K*_i_ to be 12 times lower than that of ATP, indicating a highly competitive binding of the R428 over ATP. The compound R428_1 exhibits an even lower *K*_i_ than the R428 itself, and, finally, the newly designed compound R5 shows the lowest value of *K*_i_ (2.3 nM), which is 19 times lower than that of the R428. The calculated LE for R428, R428_1, and R5 compounds are showing almost the same values; the LE for the ATP is slightly lower than for the above-mentioned compounds. Taken together, these data indicate the high efficiency of the newly designed compound R5.

**Table 7:** The inhibition constant and the ligand efficiency as calculated by the AutoDock software for the ATP, R428, best existing R428 analog—compound R428_1, and best designed compound R5—R428_1 derivative.

**Compound**	**Inhibition constant (*K*_**i**_)**	**Ligand efficiency (LE)**
ATP	566 μM	−0.14
R428	44 nM	−0.26
R428_1	31.2 nM	−0.23
R5	2.29 nM	−0.21

To validate further the molecular docking results, the free energies of binding (Δ*G*_bind_) based on the implicit solvation models were calculated for R428, its best derivative—compound R428_1, the best-designed compound R5 and ATP as a reference molecule, establishing the best ligand affinity to the Axl kinase. The MM-PBSA/GBSA calculations (Tables 8, 9), using the 50 ns MD trajectories, confirm the previous data: the compound R5 has a much higher affinity to the Axl protein in MM-PBSA (Table 8). However, the MM-GBSA test provides the same affinity to Axl for this structure as the R248 derivative form (Table 9). This outcome might be explained in such a way that the MM-PBSA model is the more “optimized,” providing the data, which are more in agreement with molecular docking experiments. This “hysteresis” phenomenon has been previously observed in the MD experiments for cyclodextrin-based complexes to assess their drug delivery potential at the blood–brain barrier (Shityakov et al., 2016a,b).

**Table 8:** Energetic analysis of the receptor–ligand complexes using the MM-PBSA implicit solvation model.

**Term (kcal/mol)**	**Axl–ATP**	**Axl–R428**	**Axl–R428_der**	**Axl–comp_R5**
Δ*E*_vdW_	−42.49	−80.51	−91.99	−98.84
Δ*E*_EL_	127.21	−2.59	−3.28	−11.55
Δ*E*_PB_	−87.06	26.65	29.59	32.79
Δ*E*_NPOLAR_	−26.03	−36.75	−40.68	−45.12
Δ*E*_DISPER_	52.22	77.39	89.66	97.81
Δ*G*_gas_	84.72	−83.09	−95.28	−110.39
Δ*G*_solv_	−60.87	67.29	78.57	85.48
Δ*G*_bind_	23.85	−15.8	−16.7	−24.91

**Table 9:** Energetic analysis of the receptor–ligand complexes using the MM-GBSA implicit solvation model.

**Term (kcal/mol)**	**Axl–ATP**	**Axl–R428**	**Axl–R428_1**	**Axl–comp_R5**
Δ*E*_vdW_	−42.49	−80.51	−91.99	−98.84
Δ*E*_EL_	127.21	−2.59	−3.28	−11.55
Δ*E*_GB_	−83.39	18.86	18.62	34.89
Δ*E*_SURF_	−5.66	−7.14	−7.74	−8.43
Δ*G*_gas_	84.72	−83.09	−95.28	−110.39
Δ*G*_solv_	−89.05	11.72	10.88	26.47
Δ*G*_bind_	−4.33	−71.38	−84.39	−83.93

On the other hand, the entropy–enthalpy compensation analysis revealed significant differences only in the Axl–ATP thermodynamics, being either an exothermic (MM-GBSA) or an endothermic (MM-PBSA) binding reaction (Table 10). Nevertheless, the experimental binding enthalpy from the previous study was found to be negative for nucleotide binding to creatine kinase (Forstner et al., 1999), indicating the possibility of an exothermic binding event for Axl–ATP. Similarly, the R428-based compounds exhibited more negative ΔH values, suggesting the enthalpy-driven binding process.

**Table 10:** Entropy–enthalpy compensation analysis of the receptor–ligand complexes from 50 ns MD simulations.

**Term (kcal/mol)**	**Axl–ATP**	**Axl–R428**	**Axl–R428_1**	**Axl–comp_R5**
*T*Δ*S* (*T* = 298.15 K)	−18.22	−18.64	−28.15	−41.98
Δ*H*^a^	−22.55	−90.02	−112.54	−125.91
Δ*H*^b^	5.63	−34.44	−44.85	−66.89

a *ΔG_MM−GBSA_ = ΔH–TΔS*.

b *ΔG_MM−PBSA_ = ΔH–TΔS*.

### Selectivity Test

To establish how selective the best-designed compound R5 toward Axl kinase is, we further performed a selectivity test against a set of other kinases such as ALK5 (Gellibert et al., 2009), ABL1 (Pemovska et al., 2015), FYN (Kinoshita et al., 2006), JAK1 (Siu et al., 2017), MET (Porter et al., 2009), Tyro3 (Powell et al., 2012), and Mer (Gajiwala et al., 2017). Table 11 reports the docking results for the compound R5 to the kinases according to the three different software—GOLD, MOE, and AutoDock. The best binding scores are highlighted in italics (see Table 11). According to the docking results, the compound R5 has shown quite good binding scores for the studied kinases, which means that the compound R5 can be further applied to target the above-mentioned kinases. Among the studied kinases, improved binding has been observed toward ABL1 and Tyro3. Interestingly, the docking of the compound R5 to the Tyro3 binding site resulted in the highest score among all kinases considered, including the primary target—the Axl kinase. This result indicates a high selectivity of the compound R5 not only toward Axl (please refer to Table 5) but also to another TAM family member—the Tyro3 kinase.

**Table 11:** The docking scores for the compound R5 against ALK5, ABL1, FYN, JAK1, MET, Tyro3, and Mer according to the GOLD, MOE, and AutoDock software.

**Kinase**	**Score (GOLD)**	**Score, kcal/mol (MOE)**	**Δ*G*, kcal/mol (AutoDock)**
ALK5	93	−8.5	−12.3
*ABL1*	*109*	*−9.0*	*−11.3*
FYN	94	−8.5	−9.0
JAK1	96	−8.8	−7.7
MET	80	−8.35	−9.7
*Tyro3*	*110*	*−9.0*	*−14.1*
Mer	95	−9.0	−7.3

*The best binding scores are indicated in italics*.

## Discussion

The expression of Axl kinase was found in various types of tumor cells (see Table 12), making this protein a promising target for novel anti-cancer pharmaceuticals. Despite the fact that there are some Axl inhibitors in the market (Myers et al., 2016), the selectivity of drugs might be a serious obstacle to overcome in order to improve their efficacy. Therefore, the novel drug-like molecules with an improved selectivity to a certain type of kinase might potentiate the therapeutic effect against malignant tumors.

**Table 12:** Expression of the Axl kinase in the different types of cancer cells.

**Cancer type**	**References**
Acute myeloid leukemia	Rochlitz et al., 1999; Hong et al., 2008
Astrocytoma	Vajkoczy et al., 2006; Keating et al., 2010
Breast cancer	Berclaz et al., 2001; Meric et al., 2002; Gjerdrum et al., 2010
Colorectal	Craven et al., 1995; Dunne et al., 2014
Esophageal adenocarcinoma	Hector et al., 2010; Hsieh et al., 2016
Glioblastoma multiforme	Hutterer et al., 2008
Gastrointestinal stromal tumor	Mahadevan et al., 2007
Gastric cancer	Wu et al., 2002
Head and neck squamous cell carcinoma	Brand et al., 2015
Hepatocellular carcinoma	Liu et al., 2016
Kaposi sarcoma	Liu et al., 2010
Lung adenocarcinoma	Shieh et al., 2005; Ishikawa et al., 2013
Melanoma	Müller et al., 2014
Oral squamous carcinoma	Lee et al., 2012
Osteosarcoma	Nakano et al., 2003
Ovarian adenocarcinoma	Chen et al., 2013; Rea et al., 2015
Pancreatic ductal adenocarcinoma	Koorstra et al., 2009
Renal cell carcinoma	Gustafsson et al., 2009
Pleural mesothelioma	Pinato et al., 2013
Urothelial carcinoma	Hattori et al., 2016
Prostate cancer	Sainaghi et al., 2005
Thyroid cancer	Ito et al., 1999
Uterine endometrial cancer	Sun et al., 2003

To follow this path, molecular docking and molecular dynamics simulation approaches were devised and implemented to compare the binding affinities of known Axl kinase inhibitors (Myers et al., 2016) and to design some novel drug candidates. We purposely did not implement here a completely new simulation strategy, but rather followed a well-validated modeling path, which has been successfully applied by experimental scientists in the design of new active compounds (Li et al., 2011; Mollard et al., 2011; Dakshanamurthy et al., 2012; Heifetz et al., 2012). It is worth to note that docking, despite its limitations, is a well-established and an experimentally approved technique for the prediction of potential active substances. For instance, Li et al. (2011) used a docking method to test 4,621 approved drugs from DrugBank against the crystal structure of MAPK14 to identify a potential anti-inflammatory drug for the treatment of chronic myeloid leukemia. The study revealed a potent inhibitor—the drug nilotinib, with an *in vitro* IC_50_ of 40 nM (Li et al., 2011). Dakshanamurthy et al. (2012) showed another successful application of computational modeling in drug discovery. In their methodology, the drug–target interaction among 3,671 FDA-approved drugs and 2,335 human protein crystal structures was predicted with 91% accuracy. In addition, Dakshanamurthy et al. discovered that the anti-parasitic drug mebendazole also revealed anti-cancer properties, with a strong inhibition activity against vascular endothelial growth factor receptor 2. The results were confirmed experimentally to the extent that effective treatment of different medulloblastoma models could be shown by applying mebendazole, including a clear impact on tumor angiogenesis (Bai et al., 2015).

Purely computational studies targeted on the understanding of agonists/antagonist interaction with a certain type of receptor for the design of new medications were performed recently. In particular, Zhu et al. (2019) applied a combination of *in silico* tools such as the 3D-QSAR, molecular docking, molecular dynamics, and free energy calculation to clarify a selectivity mechanism of glycogen synthase kinase 3β toward ATP-competitive inhibitors. They identified some key selective residues that might play an important role in the design of the novel ATP-competitive inhibitors (Zhu et al., 2019). Martínez-Campos et al. (2019) in their work employed molecular docking: firstly the QSAR method to analyze all known gamma aminobutyric acid (GABA_B_) receptor agonists and later a structure-based drug design strategy for the design of the novel compounds. They came up with six potent baclofen analogs targeted on the activation of the GABA_B_ receptor (Martínez-Campos et al., 2019). Liu et al. (2019) used virtual screening, molecular docking, and molecular dynamics tools to identify the potential methylguanine-DNA methyltransferase (MGMT) inhibitors. Their research resulted in two potent leads, the ZINC000008220033 and ZINC000001529323 compounds, which can be further optimized against the MGMT protein (Liu et al., 2019).

There are existing computational studies targeted on the development of the Axl kinase inhibitors. For instance, Fatima et al. (2017) performed a docking simulation to understand the compatibility of curcumin derivatives against a homology model of the Axl kinase active site. Similarly, Mollard et al. (2011) used an *in silico* approach to design the Axl kinase inhibitors. They also performed a homology model of the binding site for such a purpose. Moreover, these results were subsequently confirmed by experimental data (Mollard et al., 2011). In both of the above-mentioned cases, a homology model of the protein was employed. In our study, we rely instead on the experimentally solved 3D structure of the Axl kinase binding pocket (Gajiwala et al., 2017), and for more accuracy, besides the extensive molecular docking calculations, we additionally performed the MMPBSA/GBSA simulations (see Wang et al., 2019 for evaluation) to identify the best possible drug candidates. As the highest affinity toward Axl kinase was defined for the crizotinib and R428 molecules (see Table 1), they were further considered as the parental “scaffolds” for *in silico* structural modifications.

Crizotinib was already applied as a first-line therapy for the treatment of lung cancer (Awad and Shaw, 2014). This medication is also active against the ALK and hepatocyte growth factor receptor as proto-oncogene c-Met, especially in patients with the ALK-rearranged non-small cell lung cancer after oral administration (Awad and Shaw, 2014). However, most of the crizotinib-based modifications represented only a slight binding improvement to the Axl protein (Table S2; compounds C1–C10). Crizotinib and the related compounds, in line with this, work only for some time, and then there is a resistance observed in the clinic (Awad and Shaw, 2014).

**Figure 8:**
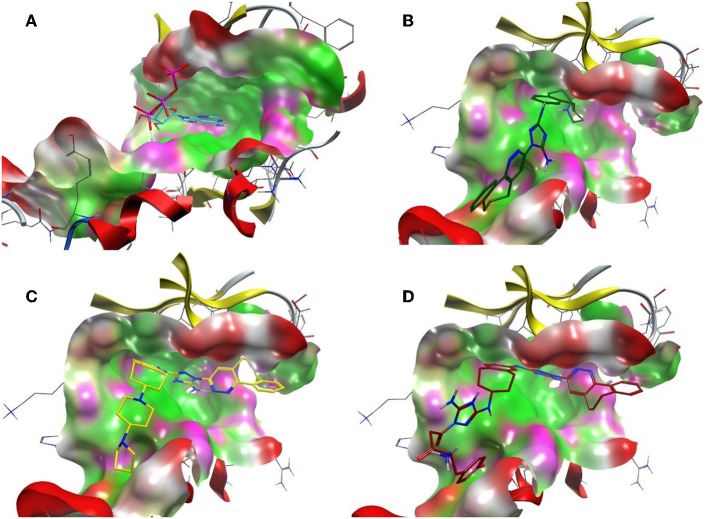
The ATP binding cavity on the surface of the Axl kinase and the drug accommodation inside the pocket. The ATP is shown in *cyan* **(A)**, the compound R428 is shown in *green* **(B)**, the compound R428_1 is shown in *yellow* **(C)**, and our best designed compound R5 is shown in *red* **(D)**. The pocket is represented as a surface, the protein chain is represented as a cartoon, the hydrophobic patches of the pocket are shown in *light green*, the hydrophilic patches are shown in *purple*, and the hydrogen bond acceptors are shown in *red*. The compounds in the pocket are represented as a licorice.

On the other hand, R428 had proven to be a selective Axl kinase inhibitor (Holland et al., 2010), with the inhibition constant in the nanomolar (nM) range (Myers et al., 2016), as well as the blocker of other Axl-associated events such as autophosphorylation, metastasis development in breast cancer and proinflammatory cytokine production (Holland et al., 2010). In addition, R428 was found to induce apoptosis in many types of cancer cells (Chen et al., 2018). Our findings concerning R428-analog-based modifications demonstrate more promising results than that of crizotinib. In particular, the designed drug candidates—compounds R3, R5, and R10 (see Table 5) —possess an improved binding property toward Axl compared to the known type I inhibitors (see Table 1). Besides this, they are also involved in the interaction with the D690, N677, M623, and H625 residues (see Table 6) that are conserved for all TAM family receptors according to the sequence alignment as shown by Gajiwala et al. (2017). To evaluate the compatibility of the compound R5 to Axl kinase, in Figure 8, for comparison, we demonstrate ATP, R428, R428_1, and R5 inside the binding pocket of Axl kinase. As the figure shows, ATP does not fit the pocket well due to its small size (see Figure 8A) and, for this reason, does not result in strong binding. R428 fits much better than ATP (see Figure 8B), and, therefore, the binding score is much higher than that of ATP. Compound R428_1, in turn, fits even better than the compound R428 (see Figure 8C), resulting in the highest score among the R428 analogs considered. Finally, our best-designed drug candidate—compound R5 (see Figure 8D)—shows the best fit and the strongest binding affinity toward kinase.

The MD simulations are in strong accordance with the molecular docking results, indicating a consistency between the results obtained by two different methodologies. In particular, the best binding molecular docking results were obtained for the R5 compound compared to the parental R428 drug and the best R428 patented analog—compound R428_1. These findings were in agreement with the MM-PBSA calculations, which were more optimized for these systems analyzed than with the MM-GBSA approach (Table 8). The latter method determined the second best affinity value for R5 to the Axl kinase pocket after Axl–R428_1 (Table 9). The discrepancies in the MM-PBSA/GBSA performances were previously emphasized in the literature, showing that MM/PBSA performed better in calculating absolute, but not always binding free energies than MM-GBSA (Hou et al., 2011). Furthermore, the calculated *K*_i_ for R5 has shown the lowest value (Table 7), indicating its high inhibition potency.

**Figure 9:**
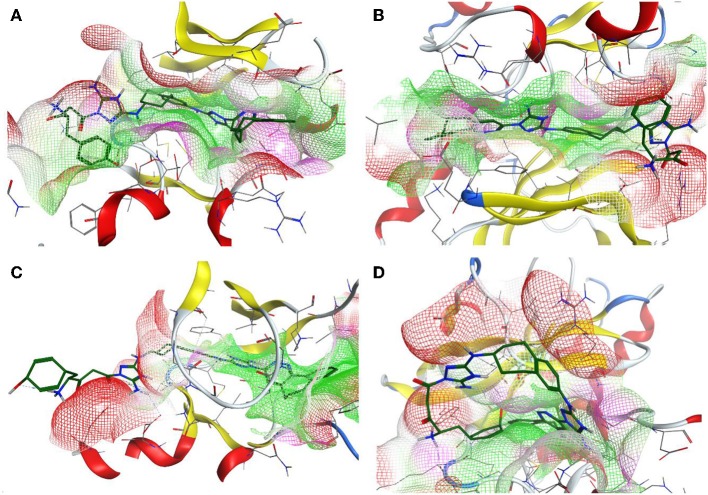
The 3D representation of the compound designed, R5, inside the binding pocket of the Axl **(A)**, Tyro3 **(B)**, ABL1 **(C)**, and MET **(D)** kinases. The pocket is shown as a van der Waals surface representation. The hydrophobic parts inside the pocket are shown in *green*, the hydrophilic parts are shown in *purple*, and the hydrogen bond acceptors are shown in *red*. The ligand is shown as a licorice representation and depicted in *forest green*.

Additionally, the molecular-docking-based selectivity test was performed by screening a set of kinases (ALK5, ABL1, FYN, JAK1, Met, Tyro3, and Mer) to evaluate the binding affinity of the compound R5 to them. Interestingly, this compound has shown the strongest binding properties toward Tyro3, which is another receptor of the TAM family. We tried to explain this phenomenon by performing a comparative analysis for the binding pockets of the high-scoring (Axl, Tyro3, and ABL1) and the relatively low-scoring (MET) kinases (see Table 11). It is clear from the molecular docking poses (see Figure 9) that R5 exhibits a “stretched” conformation inside the binding sites of Axl, Tyro3, and ABL1 and a “shrunk” form in the MET. There are four residues (D690, E546, D627, and G626) of the Axl pocket involved in the interaction with R5 (Figure S1A). Compared to the Axl pocket, five residues in the Tyro3 (K597, M596, R512, D663, and K540) and the ABL1 (D325, E329, L248, T315, and E316) pockets are involved in the interaction with R5 (see Figures S1B,C). In contrast, only three residues of the MET pocket (K1161, Y1230, and D1164) are involved in the interaction with R5 (Figure S1D). Therefore, we think that the 3D shape of the binding sites as well as the number of interacting residues influence the change in the binding scores. Furthermore, the Axl and Tyro3 kinases were already investigated as important drug targets for various types of cancers (Duan et al., 2016; Dantas-Barbosa et al., 2017), expanding the possibility of R5 application against different malignant tumors.

TAM activation can, thus, be efficiently inhibited by the small-molecule inhibitors for this interaction. For the TAM activation blocking compounds RU-301 and RU-302, Kimani et al. (2017) suggested and experimentally observed that these may act as pan-TAM inhibitors as they suppress the H1299 lung cancer tumor growth in the mouse xenograft NOND-SCIDγ model tested. Individual studies are, of course, limited to only either modeling or experiments *in vitro* or in cell culture. Similarly, inhibiting only one cancer pathway may not be the strongest strategy, particularly in view of clinical application. However, there is now consensus that, due to their well-established and clear inhibitory effect on several cancer pathways such as cell survival, invasion, migration, chemo-resistance, and metastasis, such TAM inhibitors hold great promise for cancer treatment (Davra et al., 2016). Moreover, further studies have shown that the TAM family expression often correlates with poor clinical outcomes, and there are several classes of promising TAM inhibitors according to the accumulated experimental data (Davra et al., 2016). We reveal here a particular promising TAM inhibitor derived from the experimentally validated compound R428; this is compound R5. We have, hence, good reasons, including the solid experimental validation of closely related compounds and using only well-established and experimentally validated methods and compound scoring schemes, to suggest R5 as a novel anti-cancer compound. However, this is, of course, only the starting point for further research. To be sure, this needs even more experimental data and subsequent compound-specific tests and clinical trials. As a bioinformatics group, we hope to stimulate more experimental research toward this end. Moreover, the molecular approach of the TAM-receptor inhibition implemented here is also a valuable strategy not only in cancer but also in hemostasis and thrombosis and platelet function (Law et al., 2018), such that the compound R5 should be explored and tested further and also analyzed for such indications.

### General Considerations and Caveats

It is, of course, possible to get a very high false-positive rate in the virtual screening, but this can mainly happen if you do not do any scoring or accept all targets equally well.

In particular, as the reader can see in our manuscript, the chosen strategy strongly reduces the amount of false-positives. We always start from the best possible leads, and that is not only according to the scoring but also, more importantly, according to all available experimental data.

Nevertheless, we think that it is necessary to validate the proposed compounds with actual experiments, and that is the reason why we want to publish our results here, considering other purely theory articles such as those of Fu et al. (2019), Duan et al. (2019), and Zheng et al. (2019). We acknowledge that we highly value the hard work and the final proof of a direct experiment, and the whole point of our publication is to exactly simulate this. We also have a non-trivial result, i.e., several strong pharmacological leads, including experimental validation and their actual use in clinical settings, are incorporated and now further optimized by us, yielding a top compound, among the best candidates for an important and well-known cancer target, that looks very promising by three different scoring schemes.

## Conclusion

In summary, systematic computational studies were performed to establish the best drug candidates for targeting Axl kinase to treat cancer. The first compound selection has shown that the most potent drugs for such a purpose are crizotinib, macrocyclic compound 1, and R428. Further searches were performed based on the analogs of pharmaceutically available drugs, namely, crizotinib and R428. The docking results for crizotinib analogs did not demonstrate scores higher than the original drug itself. Therefore, we focused on crizotinib by modifying it further. The R428 analog docking resulted in four high-scoring compounds, which were further utilized for structural modifications. The structural modifications of crizotinib did not show any significant Axl binding improvements, while modifications of the R428 analogs resulted in a new potential drug candidate—compound R5. Apart from the docking tests, the potency of this newly designed compound as an Axl kinase inhibitor has been confirmed by a molecular dynamics simulation in terms of protein–ligand complex stability. Furthermore, an *in silico* selectivity test against other kinases has also shown a high affinity of R5 toward ABL1 and Tyro3. Our *in silico* results suggest the application of the newly designed compound R5 particularly as an anti-cancer agent. Direct proof by experiment is now the next important step. Further synthesis and *in vitro* tests of this compound against various cancer cell lines, where Axl, ABL1, and Tyro3 play a significant role in proliferation, division and metastases are further steps to be taken, and further indications for TAM inhibition may follow.

## Data Availability Statement

All datasets generated for this study are included in the article/Supplementary Material.

## Author Contributions

ES planned the study, performed the *in silico* screening and docking of the compounds, designed the new compounds, performed a selectivity test, analyzed the data, made corresponding figures and tables, and drafted the manuscript. SS performed molecular dynamics simulation, analyzed and reported the corresponding data, made tables, and participated in the manuscript drafting and editing. TD supervised the study, analyzed its data, and participated in the manuscript drafting and editing. All authors finalized the manuscript together.

### Conflict of Interest

The authors declare that the research was conducted in the absence of any commercial or financial relationships that could be construed as a potential conflict of interest.

## References

[B1] Awad M. M. Shaw A. T. (2014). ALK inhibitors in non-small cell lung cancer: crizotinib and beyond. Clin. Adv. Hematol. Oncol. 12, 429–439. 25322323 PMC4215402

[B2] Bai R. Y. Staedtke V. Rudin C. M. Bunz F. Riggins G. J. (2015). Effective treatment of diverse medulloblastoma models with mebendazole and its impact on tumor angiogenesis. Neuro Oncol. 17, 545–554. 10.1093/neuonc/nou234 25253417 PMC4483072

[B3] Baldi A. (2010). Computational approaches for drug design and discovery: an overview. Syst. Rev. Pharm. 1:99 10.4103/0975-8453.59519

[B4] Berclaz G. Altermatt H. J. Rohrbach V. Kieffer I. Dreher E. Andres A. C. (2001). Estrogen dependent expression of the receptor tyrosine kinase axl in normal and malignant human breast. Ann. Oncol. 12, 819–24. 10.1023/a:1011126330233 11484958

[B5] Blume-Jensen P. Hunter T. (2001). Oncogenic kinase signalling. Nature 411, 355–365. 10.1038/35077225 11357143

[B6] Brand T. M. Iida M. Stein A. P. Corrigan K. L. Braverman C. M. Coan J. P.. (2015). AXL is a logical molecular target in head and neck squamous cell carcinoma. Clin. Cancer Res. 21, 2601–2612. 10.1158/1078-0432.CCR-14-2648 25767293 PMC5032632

[B7] Case D. A. Cheatham T. E. Darden T. Gohlke H. Luo R. Merz K. M.. (2005). The Amber biomolecular simulation programs. J. Comput. Chem. 26, 1668–1688. 10.1002/jcc.20290 16200636 PMC1989667

[B8] Chan S. L. Labute P. (2010). Training a scoring function for the alignment of small molecules. J. Chem. Inf. Model. 50, 1724–1735. 10.1021/ci100227h 20831240 PMC2946173

[B9] Chen F. Song Q. Yu Q. (2018). Axl inhibitor R428 induces apoptosis of cancer cells by blocking lysosomal acidification and recycling independent of Axl inhibition. Am. J. Cancer Res. 8, 1466–1482. 30210917 PMC6129480

[B10] Chen P. X. Li Q. Y. Yang Z. (2013). Axl and prostasin are biomarkers for prognosis of ovarian adenocarcinoma. Ann. Diagn. Pathol. 17, 425–429. 10.1016/j.anndiagpath.2013.01.005 23707658

[B11] Craven R. J. Xu L. Weiner T. M. Fridell Y.-W. Dent G. A. Srivastava S.. (1995). Receptor tyrosine kinases expressed in metastatic colon cancer. Int. J. Cancer 60, 791–797. 10.1002/ijc.2910600611 7896447

[B12] Dakshanamurthy S. Issa N. T. Assefnia S. Seshasayee A. Peters O. J. Madhavan S.. (2012). Predicting new indications for approved drugs using a proteochemometric method. J. Med. Chem. 55, 6832–6848. 10.1021/jm300576q 22780961 PMC3419493

[B13] Dantas-Barbosa C. Lesluyes T. Loarer F. L. Chibon F. Treilleux I. Coindre J. M.. (2017). Expression and role of TYRO3 and AXL as potential therapeutical targets in leiomyosarcoma. Br. J. Cancer 117, 1787–1797. 10.1038/bjc.2017.354 29024938 PMC5729471

[B14] Davra V. Kimani S. G. Calianese D. Birge R. B. (2016). Ligand activation of TAM family receptors—implications for tumor biology and therapeutic response. Cancers 8:107. 10.3390/cancers8120107 27916840 PMC5187505

[B15] Duan L. Guo X. Cong Y. Feng G. Li Y. Zhang J. Z. H. (2019). Accelerated molecular dynamics simulation for helical proteins folding in explicit water. Front Chem. 7:540. 10.3389/fchem.2019.00540 31448259 PMC6691143

[B16] Duan Y. Wong W. Chua S. C. Wee H. L. Lim S. G. Chua B. T.. (2016). Overexpression of Tyro3 and its implications on hepatocellular carcinoma progression. Int. J. Oncol. 48, 358–366. 10.3892/ijo.2015.3244 26573872

[B17] Dunne P. D. McArt D. G. Blayney J. K. Kalimutho M. Greer S. Wang T.. (2014). AXL is a key regulator of inherent and chemotherapy-induced invasion and predicts a poor clinical outcome in early-stage colon cancer. Clin. Cancer Res. 20, 164–175. 10.1158/1078-0432.CCR-13-1354 24170546 PMC3885388

[B18] Essmann U. Perera L. Berkowitz M. L. Darden T. Lee H. Pedersen L. G. (1995). A smooth particle mesh Ewald method. J. Chem. Phys. 103, 8577–8593. 10.1063/1.470117

[B19] Fatima G. Loubna A. Wiame L. Azeddine I. (2017). *In silico* inhibition studies of AXL kinase by curcumin and its natural derivatives. J. Appl. Bioinforma. Comput. Biol. 6:3 10.4172/2329-9533.1000142

[B20] Forli S. Huey R. Pique M. E. Sanner M. F. Goodsell D. S. Olson A. J. (2016). Computational protein-ligand docking and virtual drug screening with the AutoDock suite. Nat. Protoc. 11, 905–919. 10.1038/nprot.2016.051 27077332 PMC4868550

[B21] Forstner M. Berger C. Wallimann T. (1999). Nucleotide binding to creatine kinase: an isothermal titration microcalorimetry study. FEBS Lett. 461, 111–114. 10.1016/s0014-5793(99)01431-3 10561506

[B22] Fu Y. Liu Y. X. Yi K. H. Li M. Q. Li J. Z. Ye F. (2019). Quantitative structure–activity relationship studies and molecular dynamics simulations of 2-(aryloxyacetyl)cyclohexane-1,3-diones derivatives as 4-hydroxyphenylpyruvate dioxygenase inhibitors. Front Chem. 7:556. 10.3389/fchem.2019.00556 31482084 PMC6710436

[B23] Gajiwala K. S. Grodsky N. Bolaños B. Feng J. Ferre R. A. Timofeevski S.. (2017). The Axl kinase domain in complex with a macrocyclic inhibitor offers first structural insights into an active TAM receptor kinase. J. Biol. Chem. 292, 15705–15716. 10.1074/jbc.M116.771485 28724631 PMC5612104

[B24] Gellibert F. Fouchet M.-H. Nguyen V.-L. Wang R. Krysa G. de Gouville A.-C.. (2009). Design of novel quinazoline derivatives and related analogues as potent and selective ALK5 inhibitors. Bioorg. Med. Chem. Lett. 19, 2277–2281. 10.1016/j.bmcl.2009.02.087 19285388

[B25] Gjerdrum C. Tiron C. Høiby T. Stefansson I. Haugen H. Sandal T.. (2010). Axl is an essential epithelial-to-mesenchymal transition-induced regulator of breast cancer metastasis and patient survival. Proc. Natl. Acad. Sci. U.S.A. 107, 1124–1129. 10.1073/pnas.0909333107 20080645 PMC2824310

[B26] Gustafsson A. Martuszewska D. Johansson M. Ekman C. Hafizi S. Ljungberg B.. (2009). Differential expression of Axl and Gas6 in renal cell carcinoma reflecting tumor advancement and survival. Clin. Cancer Res. 15, 4742–4749. 10.1158/1078-0432.CCR-08-2514 19567592

[B27] Han J. Tian R. Yong B. Luo C. Tan P. Shen J.. (2013). Gas6/Axl mediates tumor cell apoptosis, migration and invasion and predicts the clinical outcome of osteosarcoma patients. Biochem. Biophys. Res. Commun. 435, 493–500. 10.1016/j.bbrc.2013.05.019 23684620

[B28] Hasanbasic I. Rajotte I. Blostein M. (2005). The role of γ-carboxylation in the anti-apoptotic function of gas6. J. Thromb. Haemost. 3, 2790–2797. 10.1111/j.1538-7836.2005.01662.x 16359517

[B29] Hattori S. Kikuchi E. Kosaka T. Miyazaki Y. Tanaka N. Miyajima A.. (2016). Relationship between increased expression of the Axl/Gas6 signal cascade and prognosis of patients with upper tract urothelial carcinoma. Ann. Surg. Oncol. 23, 663–670. 10.1245/s10434-015-4848-x 26350366

[B30] Hector A. Montgomery E. A. Karikari C. Canto M. Dunbar K. B. Wang J. S.. (2010). The Axl receptor tyrosine kinase is an adverse prognostic factor and a therapeutic target in esophageal adenocarcinoma. Cancer Biol. Ther. 10, 1009–1018. 10.4161/cbt.10.10.13248 20818175 PMC3025816

[B31] Heifetz A. Morris G. B. Biggin P. C. Barker O. Fryatt T. Bentley J.. (2012). Study of human orexin-1 and−2 G-protein-coupled receptors with novel and published antagonists by modeling, molecular dynamics simulations, and site-directed mutagenesis. Biochemistry 51, 3178–3197. 10.1021/bi300136h 22448975

[B32] Holland S. J. Pan A. Franci C. Hu Y. Chang B. Li W.. (2010). R428, a selective small molecule inhibitor of Axl kinase, blocks tumor spread and prolongs survival in models of metastatic breast cancer. Cancer Res. 70, 1544–1554. 10.1158/0008-5472.CAN-09-2997 20145120

[B33] Hong C. C. Lay J. D. Huang J. S. Cheng A. L. Tang J. L. Lin M. T.. (2008). Receptor tyrosine kinase AXL is induced by chemotherapy drugs and overexpression of AXL confers drug resistance in acute myeloid leukemia. Cancer Lett. 268, 314–324. 10.1016/j.canlet.2008.04.017 18502572

[B34] Hou T. Wang J. Li Y. Wang W. (2011). Assessing the performance of the MM/PBSA and MM/GBSA methods. 1. The accuracy of binding free energy calculations based on molecular dynamics simulations. J. Chem. Inf. Model. 51, 69–82. 10.1021/ci100275a 21117705 PMC3029230

[B35] Hsieh M. S. Yang P. W. Wong L. F. Lee J. M. (2016). The AXL receptor tyrosine kinase is associated with adverse prognosis and distant metastasis in esophageal squamous cell carcinoma. Oncotarget 7, 36956–36970. 10.18632/oncotarget.9231 27172793 PMC5095051

[B36] Hutterer M. Knyazev P. Abate A. Reschke M. Maier H. Stefanova N.. (2008). Axl and growth arrest-specific gene 6 are frequently overexpressed in human gliomas and predict poor prognosis in patients with glioblastoma multiforme. Clin. Cancer Res. 14, 130–138. 10.1158/1078-0432.CCR-07-0862 18172262

[B37] Ishikawa M. Sonobe M. Nakayama E. Kobayashi M. Kikuchi R. Kitamura J.. (2013). Higher expression of receptor tyrosine kinase axl, and differential expression of its ligand, gas6, predict poor survival in lung adenocarcinoma patients. Ann. Surg. Oncol. 20, S467–S476. 10.1245/s10434-012-2795-3 23242819 PMC3853411

[B38] Ito T. Ito M. Naito S. Ohtsuru A. Nagayama Y. Kanematsu T.. (1999). Expression of the Axl receptor tyrosine kinase in human thyroid carcinoma. Thyroid 9, 563–567. 10.1089/thy.1999.9.563 10411118

[B39] Jones G. Willett P. Glen R. C. Leach A. R. Taylor R. (1997). Development and validation of a genetic algorithm for flexible docking. J. Mol. Biol. 267, 727–748. 10.1006/jmbi.1996.0897 9126849

[B40] Keating A. K. Kim G. K. Jones A. E. Donson A. M. Ware K. Mulcahy J. M.. (2010). Inhibition of Mer and Axl receptor tyrosine kinases in astrocytoma cells leads to increased apoptosis and improved chemosensitivity. Mol. Cancer Ther. 9, 1298–1307. 10.1158/1535-7163.MCT-09-0707 20423999 PMC3138539

[B41] Kim S. Thiessen P. A. Bolton E. E. Chen J. Fu G. Gindulyte A.. (2016). PubChem substance and compound databases. Nucleic Acids Res. 44, D1202–D1213. 10.1093/nar/gkv951 26400175 PMC4702940

[B42] Kimani S. G. Kumar S. Bansal N. Singh K. Kholodovych V. Comollo T.. (2017). Small molecule inhibitors block Gas6-inducible TAM activation and tumorigenicity. Sci Rep. 7:43908. 10.1038/srep43908 28272423 PMC5341070

[B43] Kinoshita T. Matsubara M. Ishiguro H. Okita K. Tada T. (2006). Structure of human Fyn kinase domain complexed with staurosporine. Biochem. Biophys. Res. Commun. 346, 840–844. 10.1016/j.bbrc.2006.05.212 16782058

[B44] Kollman P. A. Massova I. Reyes C. Kuhn B. Huo S. Chong L.. (2000). Calculating structures and free energies of complex molecules: combining molecular mechanics and continuum models. Acc. Chem. Res. 33, 889–897. 10.1021/ar000033j 11123888

[B45] Koorstra J. B. Karikari C. A. Feldmann G. Bisht S. Rojas P. L. Offerhaus G. J.. (2009). The Axl receptor tyrosine kinase confers an adverse prognostic influence in pancreatic cancer and represents a new therapeutic target. Cancer Biol. Ther. 8, 618–626. 10.4161/cbt.8.7.7923 19252414 PMC2678175

[B46] Law L. A. Graham D. K. Di Paola J. Branchford B. R. (2018). GAS6/TAM pathway signaling in hemostasis and thrombosis. Front Med. 5:137. 10.3389/fmed.2018.00137 29868590 PMC5954114

[B47] Lee C. H. Yen C. Y. Liu S. Y. Chen C. K. Chiang C. F. Shiah S. G.. (2012). Axl is a prognostic marker in oral squamous cell carcinoma. Ann. Surg. Oncol. 19, 500–508. 10.1245/s10434-011-1985-8 21842265

[B48] Lemke G. Rothlin C. V. (2008). Immunobiology of the TAM receptors. Nat. Rev. Immunol. 8, 327–336. 10.1038/nri2303 18421305 PMC2856445

[B49] Li Y. Ye X. Tan C. Hongo J. A. Zha J. Liu J.. (2009). Axl as a potential therapeutic target in cancer: role of Axl in tumor growth, metastasis and angiogenesis. Oncogene 28, 3442–3455. 10.1038/onc.2009.212 19633687

[B50] Li Y. Y. An J. Jones S. J. M. (2011). A computational approach to finding novel targets for existing drugs. PLoS Comput. Biol. 7:e1002139. 10.1371/journal.pcbi.1002139 21909252 PMC3164726

[B51] Liu J. Wang K. Yan Z. Xia Y. Li J. Shi L.. (2016). Axl expression stratifies patients with poor prognosis after hepatectomy for hepatocellular carcinoma. PLoS ONE 11:e0154767. 10.1371/journal.pone.0154767 27182739 PMC4868325

[B52] Liu R. Gong M. Li X. Zhou Y. Gao W. Tulpule A.. (2010). Induction, regulation, and biologic function of Axl receptor tyrosine kinase in Kaposi sarcoma. Blood 116, 297–305. 10.1182/blood-2009-12-257154 20442363 PMC2910613

[B53] Liu Y. Li W. Zhao Y. Zhong S. Wang X Jiang S. (2019). Computational study on novel natural inhibitors targeting O6-methylguanine-DNA methyltransferase (MGMT). World Neurosurg. 130, e294–e306. 10.1016/j.wneu.2019.08.046 31203065

[B54] Mahadevan D. Cooke L. Riley C. Swart R. Simons B. Della Croce K.. (2007). A novel tyrosine kinase switch is a mechanism of imatinib resistance in gastrointestinal stromal tumors. Oncogene 26, 3909–3919. 10.1038/sj.onc.1210173 17325667

[B55] March-Vila E. Pinzi L. Sturm N. Tinivella A. Engkvist O. Chen H.. (2017). On the integration of *in silico* drug design methods for drug repurposing. Front. Pharmacol. 8:298. 10.3389/fphar.2017.00298 28588497 PMC5440551

[B56] Martínez-Campos Z. Pastor N. Pineda-Urbina K. Gómez-Sandoval Z. Mario Fernández-Zertuche R. S. (2019). *In silico* structure-based design of GABAB receptor agonists using a combination of docking and QSAR. Chem. Biol. Drug Des. 94, 1782–1798. 10.1111/cbdd.13580 31207116

[B57] Meric F. Lee W.-P. Sahin A. Zhang H. Kung H.-J. Hung M.-C. (2002). Expression profile of tyrosine kinases in breast cancer. Clin. Cancer Res. 8, 361–367. 11839650

[B58] Miyamoto S. Kollman P. A. (1992). Settle: an analytical version of the SHAKE and RATTLE algorithm for rigid water models. J. Comput. Chem. 13, 952–962. 10.1002/jcc.540130805

[B59] Molecular Operating Environment (MOE) (2016). Molecular Operating Environment (MOE), 2013.08. Montreal, QC: Chemical Computing Group Inc.

[B60] Mollard A. Warner S. L. Call L. T. Wade M. L. Bearss J. J. Verma A.. (2011). Design, synthesis, and biological evaluation of a series of novel AXL kinase inhibitors. ACS Med. Chem. Lett. 2, 907–912. 10.1021/ml200198x 22247788 PMC3254106

[B61] Morris G. M. Huey R. Lindstrom W. Sanner M. F. Belew R. K. Goodsell D. S. (2009). AutoDock4 and AutoDockTools4: automated docking with selective receptor flexibility. J. Comput. Chem. 30, 2785–2791. 10.1002/jcc.21256 19399780 PMC2760638

[B62] Müller J. Krijgsman O. Tsoi J. Robert L. Hugo W. Song C.. (2014). Low MITF/AXL ratio predicts early resistance to multiple targeted drugs in melanoma. Nat. Commun. 5:5712. 10.1038/ncomms6712 25502142 PMC4428333

[B63] Myers S. H. Brunton V. G. Unciti-Broceta A. (2016). AXL inhibitors in cancer: a medicinal chemistry perspective. J. Med. Chem. 59, 3593–3608. 10.1021/acs.jmedchem.5b01273 26555154

[B64] Nakano T. Tani M. Ishibashi Y. Kimura K. Park Y.-B. Imaizumi N.. (2003). Biological properties and gene expression associated with metastatic potential of human osteosarcoma. Clin. Exp. Metastasis 20, 665–74. 10.1023/a:1027355610603 14669798

[B65] O'Bryan J. P. Frye R. A. Cogswell P. C. Neubauer A. Kitch B. Prokop C.. (1991). Axl, a transforming gene isolated from primary human myeloid leukemia cells, encodes a novel receptor tyrosine kinase. Mol. Cell. Biol. 11, 5016–5031. 10.1128/mcb.11.10.5016 1656220 PMC361494

[B66] Ou-Yang S. S. Lu J. Y. Kong X. Q. Liang Z. J. Luo C. Jiang H. (2012). Computational drug discovery. Acta Pharmacol. Sin. 33, 1131–1140. 10.1038/aps.2012.109 22922346 PMC4003107

[B67] Pemovska T. Johnson E. Kontro M. Repasky G. A. Chen J. Wells P.. (2015). Axitinib effectively inhibits BCR-ABL1(T315I) with a distinct binding conformation. Nature 519, 102–105. 10.1038/nature14119 25686603

[B68] Pettersen E. F. Goddard T. D. Huang C. C. Couch G. S. Greenblatt D. M. Meng E. C.. (2004). UCSF chimera—a visualization system for exploratory research and analysis. J. Comput. Chem. 25, 1605–1612. 10.1002/jcc.20084 15264254

[B69] Pinato D. J. Mauri F. A. Lloyd T. Vaira V. Casadio C. Boldorini R. L.. (2013). The expression of Axl receptor tyrosine kinase influences the tumour phenotype and clinical outcome of patients with malignant pleural mesothelioma. Br. J. Cancer 108, 621–628. 10.1038/bjc.2013.9 23361052 PMC3593571

[B70] Porter J. Lumb S. Franklin R. J. Gascon-Simorte J. M. Calmiano M. Riche K.. (2009). Discovery of 4-azaindoles as novel inhibitors of c-Met kinase. Bioorganic Med. Chem. Lett. 19, 2780–2784. 10.1016/j.bmcl.2009.03.110 19369077

[B71] Powell N. A. Kohrt J. T. Filipski K. J. Kaufman M. Sheehan D. Edmunds J. E.. (2012). Novel and selective spiroindoline-based inhibitors of sky kinase. Bioorg. Med. Chem. Lett. 22, 190–193. 10.1016/j.bmcl.2011.11.036 22119469

[B72] Quong R. Y. Bickford S. T. Ing Y. L. Terman B. Herlyn M. Lassam N. J. (1994). Protein kinases in normal and transformed melanocytes. Melanoma Res. 4, 313–319. 10.1097/00008390-199410000-00008 7858416

[B73] Rankin E. B. Giaccia A. J. (2016). The receptor tyrosine kinase AXL in cancer progression. Cancers. 8:103. 10.3390/cancers8110103 27834845 PMC5126763

[B74] Rea K. Pinciroli P. Sensi M. Alciato F. Bisaro B. Lozneanu L.. (2015). Novel Axl-driven signaling pathway and molecular signature characterize high-grade ovarian cancer patients with poor clinical outcome. Oncotarget 6, 30859–30875. 10.18632/oncotarget.5087 26356564 PMC4741573

[B75] Robinson D. R. Wu Y. M. Lin S. F. (2000). The protein tyrosine kinase family of the human genome. Oncogene 19, 5548–5557. 10.1038/sj.onc.1203957 11114734

[B76] Rochlitz C. Lohri A. Bacchi M. Schmidt M. Nagel S. Fopp M.. (1999). Axl expression is associated with adverse prognosis and with expression of Bcl-2 and CD34 in de novo acute myeloid leukemia (AML): results from a multicenter trial of the Swiss Group for Clinical Cancer Research (SAKK). Leukemia 13, 1352–1358. 10.1038/sj.leu.2401484 10482985

[B77] Roskoski R. (2016). Classification of small molecule protein kinase inhibitors based upon the structures of their drug–enzyme complexes. Pharmacol. Res. 103, 26–48. 10.1016/j.phrs.2015.10.021 26529477

[B78] Sainaghi P. P. Castello L. Bergamasco L. Galletti M. Bellosta P. Avanzi G. C. (2005). Gas6 induces proliferation in prostate carcinoma cell lines expressing the Axl receptor. J. Cell Physiol. 204, 36–44. 10.1002/jcp.20265 15605394

[B79] Sasaki T. Knyazev P. G. Clout N. J. Cheburkin Y. Göhring W. Ullrich A.. (2006). Structural basis for Gas6-Axl signalling. EMBO J. 25, 80–87. 10.1038/sj.emboj.7600912 16362042 PMC1356355

[B80] Ségaliny A. I. Tellez-Gabriel M. Heymann M. F. Heymann D. (2015). Receptor tyrosine kinases: characterisation, mechanism of action and therapeutic interests for bone cancers. J. Bone Oncol. 4, 1–12. 10.1016/j.jbo.2015.01.001 26579483 PMC4620971

[B81] Shieh Y. S. Lai C. Y. Kao Y. R. Shiah S. G. Chu Y. W. Lee H. S.. (2005). Expression of Axl in lung adenocarcinoma and correlation with tumor progression. Neoplasia 7, 1058–1064. 10.1593/neo.05640 16354588 PMC1501169

[B82] Shityakov S. Roewer N. Förster C. Broscheit J.-A. (2017). *In silico* investigation of propofol binding sites in human serum albumin using explicit and implicit solvation models. Comput. Biol. Chem. 70, 191–197. 10.1016/j.compbiolchem.2017.06.004 28917201

[B83] Shityakov S. Salmas R. E. Durdagi S. Salvador E. Pápai K. Yáñez-Gascón M. J.. (2016a). Characterization, *in vivo* evaluation, and molecular modeling of different propofol–cyclodextrin complexes to assess their drug delivery potential at the blood–brain barrier level. J. Chem. Inf. Model. 56, 1914–1922. 10.1021/acs.jcim.6b00215 27589557

[B84] Shityakov S. Salmas R. E. Salvador E. Roewer N. Broscheit J. Förster C. (2016b). Evaluation of the potential toxicity of unmodified and modified cyclodextrins on murine blood–brain barrier endothelial cells. J. Toxicol. Sci. 41, 175–184. 10.2131/jts.41.175 26961601

[B85] Siu T. Brubaker J. Fuller P. Torres L. Zeng H. Close J. (2017). The discovery of 3-((4-chloro-3-methoxyphenyl)amino)-1-((3R,46S)-4-cyanotetrahydro-2H-pyran-3-yl)-1H-pyrazole-4-carboxamide, a highly ligand efficient and efficacious janus kinase 1 selective inhibitor with favorable pharmacokinetic properties. J. Med. Chem. 60, 9676–9690. 10.1021/acs.jmedchem.7b01135 29156136

[B86] Solomon B. J. Mok T. Kim D.-W. Wu Y.-L. Nakagawa K. Mekhail T.. (2014). First-line crizotinib versus chemotherapy in *ALK*-positive lung cancer. N. Engl. J. Med. 371, 2167–2177. 10.1056/NEJMoa1408440 25470694

[B87] Stitt T. N. Conn G. Goret M. Lai C. Bruno J. Radzlejewski C.. (1995). The anticoagulation factor protein S and its relative, Gas6, are ligands for the Tyro 3/Axl family of receptor tyrosine kinases. Cell 80, 661–670. 10.1016/0092-8674(95)90520-0 7867073

[B88] Sun W. S. Fujimoto J. Tamaya T. (2003). Clinical implications of coexpression of growth arrest-specific gene 6 and receptor tyrosine kinases Axl and sky in human uterine leiomyoma. Mol. Hum. Reprod. 9, 701–707. 10.1093/molehr/gag082 14561812

[B89] Vajkoczy P. Knyazev P. Kunkel A. Capelle H. H. Behrndt S. von Tengg-Kobligk H.. (2006). Dominant-negative inhibition of the Axl receptor tyrosine kinase suppresses brain tumor cell growth and invasion and prolongs survival. Proc. Natl. Acad. Sci. U.S.A. 103, 5799–5804. 10.1073/pnas.0510923103 16585512 PMC1458653

[B90] Varnum B. C. Young C. Elliott G. Garcia A. Bartley T. D. Fridell Y. W.. (1995). Axl receptor tyrosine kinase stimulated by the vitamin K-dependent protein encoded by growth-arrest-specific gene 6. Nature 373, 623–626. 10.1038/373623a0 7854420

[B91] Wang E Sun H. Wang J. Wang Z Liu H Zhang J. Z. H (2019). End-point binding free energy calculation with MM/PBSA and MM/GBSA: strategies and application in drug design. Chem. Rev. 119, 9478–9508. 10.1021/acs.chemrev.9b00055 31244000

[B92] Wu C. W. Li A. F. Chi C. W. Lai C. H. Huang C. L. Lo S. S.. (2002). Clinical significance of AXL kinase family in gastric cancer. Anticancer Res. 22, 1071–1078. 12168903

[B93] Wu P. Clausen M. H. Nielsen T. E. (2015). Allosteric small-molecule kinase inhibitors. Pharmacol. Ther. 156, 59–68. 10.1016/j.pharmthera.2015.10.002 26478442

[B94] Zhang J. Yang P. L. Gray N. S. (2009). Targeting cancer with small molecule kinase inhibitors. Nat. Rev. Cancer 9, 28–39. 10.1038/nrc2559 19104514 PMC12406740

[B95] Zhang Y.-X. Knyazev P. G. Cheburkin Y. V. Sharma K. Knyazev Y. P. Orfi L.. (2008). AXL is a potential target for therapeutic intervention in breast cancer progression. Cancer Res. 68, 1905–1915. 10.1158/0008-5472.CAN-07-2661 18339872

[B96] Zheng L. Xia K. Mu Y. (2019). Ligand binding induces agonistic-like conformational adaptations in helix 12 of progesterone receptor ligand binding domain. Front Chem. 7:315. 10.3389/fchem.2019.00315 31134186 PMC6514052

[B97] Zhu J. Wu Y. Xu L., J. J (2019). Theoretical studies on the selectivity mechanisms of glycogen synthase kinase 3β (GSK3β) with pyrazine ATP-competitive inhibitors by 3D-QSAR, molecular docking, molecular dynamics simulation and free energy calculations. Curr Comput Aided Drug Des. 16, 17–30. 10.2174/1573409915666190708102459 PMC6967214 31284868

