# Supplementary material for: Rational Drug Design of Axl Tyrosine Kinase Type I Inhibitors as Promising Candidates Against Cancer
Source: Front Chem. 2020 Feb 4;7:920. doi: 10.3389/fchem.2019.00920 (PMC7010640; doi:10.3389/fchem.2019.00920)
Supplement: Supplementary file 1 [file Data_Sheet_1.pdf]

Supplementary Material

Contents:

Table S1. Docking scores according to GOLD, MOE and AutoDock for modifications of R428_2, R428_3 and R428_4 compounds

Table S2. Docking scores according to GOLD, MOE and AutoDock for the new designed compounds from Crizotinib.

Figure S1. 2D interaction diagrams of designed compound R5 with the binding pockets of Axl, Tyro3, ABL1 and Met kinases.

PubChem CIDs for R428 patented analogs

PubChem CIDs for Crizotinib patented analogs

Table S1. Docking scores according to GOLD, MOE and AutoDock for modifications of R428_2, R428_3 and R428_4 compounds.[1]

[1]The modifications of R428_2, R428_3 and R428_4 corresponding to the following PubChem CIDs: 67104254, 67106757 and 67103760, respectively.

**Compound No.**	**2D Structure**	**Score (GOLD)**	**Score, kcal/mol (MOE)**	**ΔG, kcal/mol**
R1’	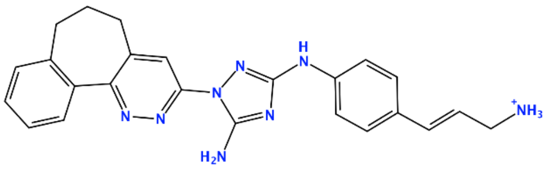	67	-6.42	-9.99
R2’	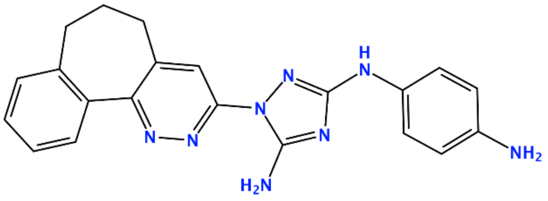	57	-6.71	-8.56
R3’	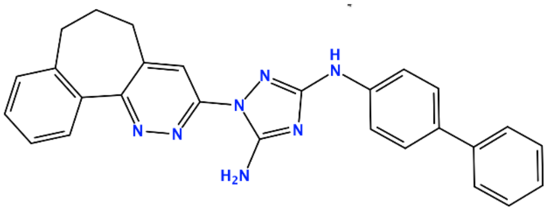	67	-7.10	-10.24
R4’	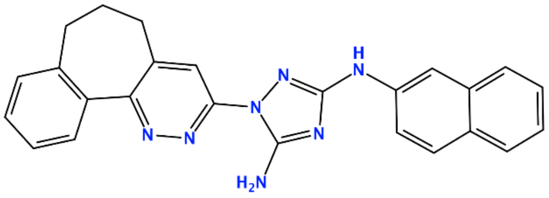	65.2	-7.17	-11.49

R5’	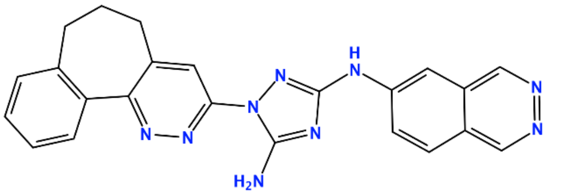	58.55	-6.97	-10.84
R6’	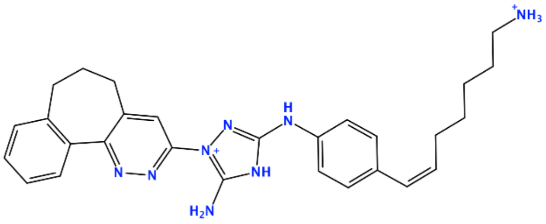	83.3	-7.61	-9.11
R7’	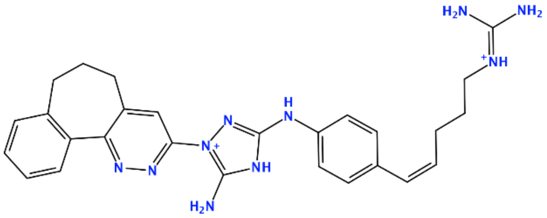	83.46	-7.77	-8.94
R8’	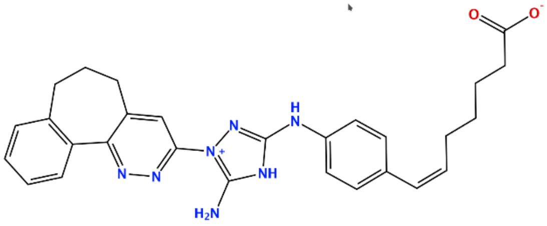	82.35	-7.41	-7.76
R9’	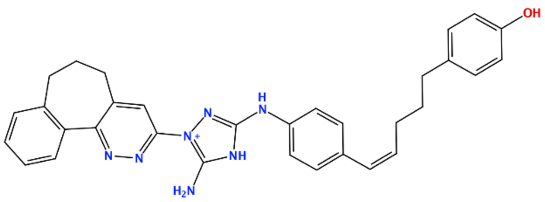	85.27	-8.01	-9.92
R10’	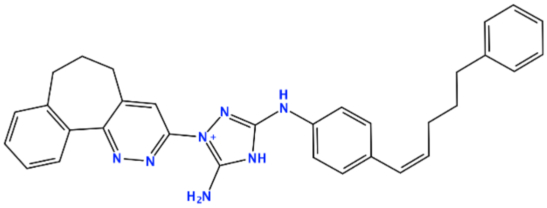	85.28	-8.29	-10.34

R11’	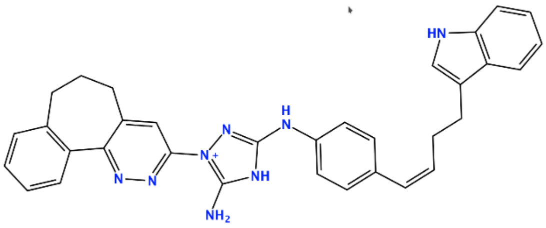	84.75	-7.52	-9.76

Table S2. Docking scores according to GOLD, MOE and Autodock for the new designed compounds from Crizotinib.				
**Compound No.**	**2D Structure**	**Score (GOLD)**	**Score, kcal/mol (MOE)**	**ΔG, kcal/mol**
C1	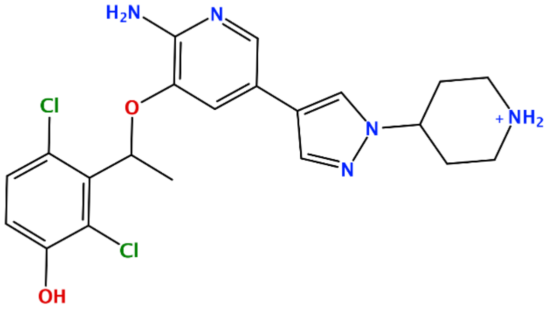	62.04	-7.41	-9.32
C2	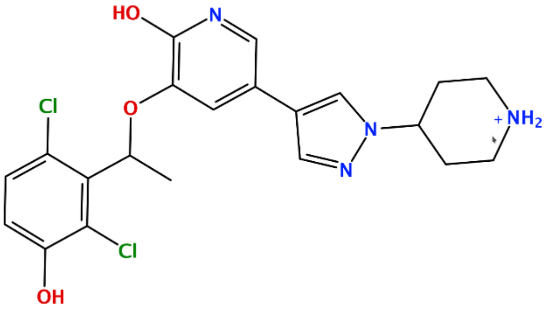	64.33	-7.67	-9.82

C3	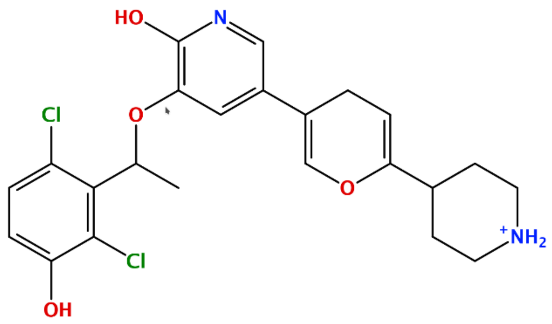	59.25	-7.05	-9.39
C4	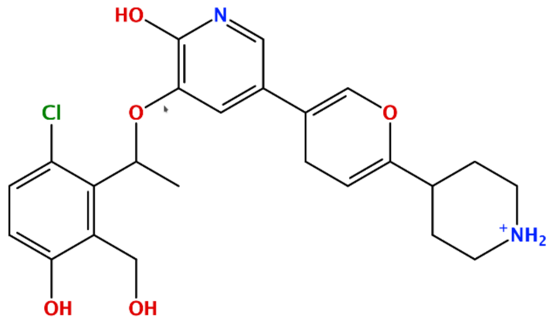	65.78	-6.9	-8.92
C5	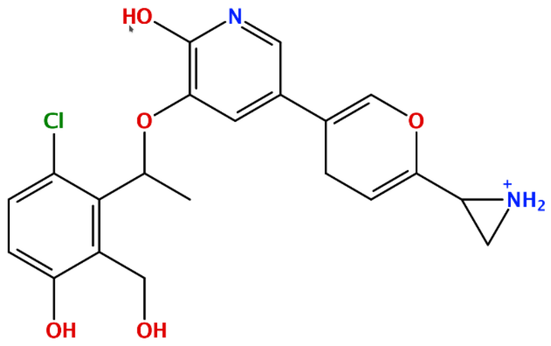	60.34	-7.24	-8.62
C6	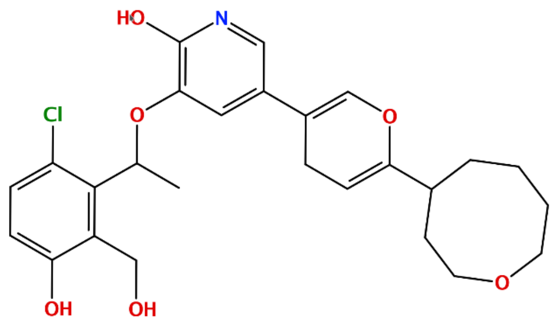	62.75	-7.97	-8.27

C7	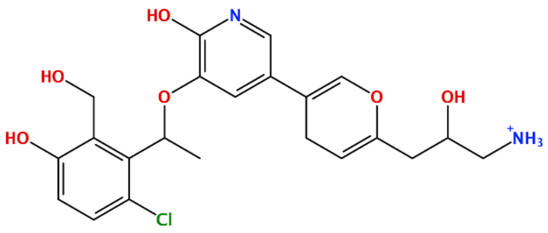	63.06	-7.21	-8.09
C8	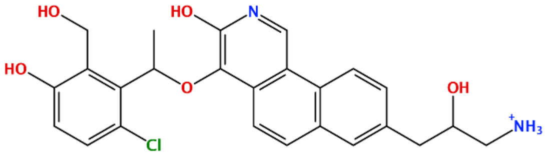	63.41	-7.22	-8.76
C9	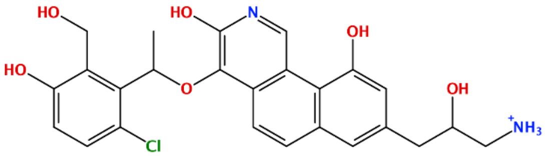	62.09	-7.23	-8.66
C10	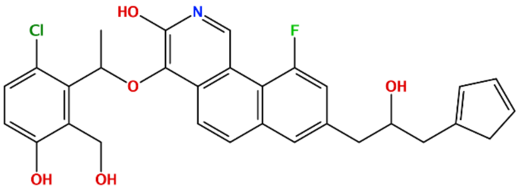	68.35	-7.44	-8.46

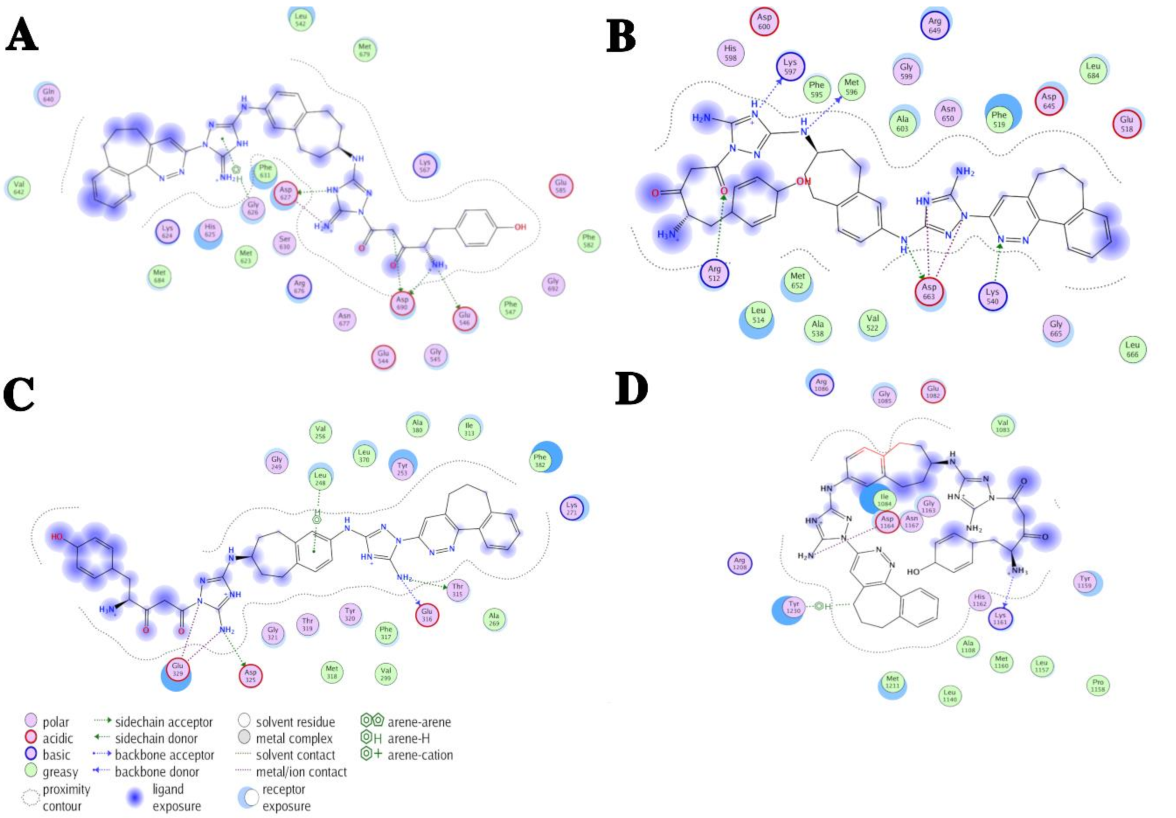

Figure S1. 2D interaction diagrams of designed compound R5 with the binding pockets of Axl (A), Tyro3 (B), ABL1 (C) and Met (D) kinases.

PubChem CIDs for R428 patented analogs:

25123113, 25123764, 25123765, 25123766, 25124093, 25124412, 25124413, 25124416, 25124748, 25126438, 25126441, 25126768, 25126769, 25126770, 25126771, 25127082, 25127084, 25127085, 25127087, 25127411, 25127412, 25127413, 25127729, 25127732, 25128065, 25242518, 44555405, 44608272, 44608274, 44608478, 46843554, 46843555, 46843556, 46843635, 46843636, 46843709, 46843710, 46843711, 46843712, 46843713, 46843779, 46843780, 46843781, 46843782, 46843783, 46843846, 46843849, 46843914, 46843915, 46843916, 46843917, 46843983, 46843984, 46843985, 46844040, 46844043, 46844099, 46844156, 46844158, 46844160, 58247200, 59276159, 66694799, 66694824, 66694833, 66694842, 66694868, 66694872, 66694875, 66694882, 66694895, 66694908, 66694911, 66694913, 66694927, 66694944, 66694947,66694949, 66694955, 66695119, 66695125, 66695128, 37103757, 67103760, 67103793, 67103828, 67103831, 67103892, 67103902, 67103980,

67103984, 67104015, 67104024, 67104046, 67104110, 67104115, 67104119, 67104124, 67104128, 67104199, 67104235, 67104240, 67104245, 67104254, 67104256, 67104272, 67104274, 67104292, 67104295, 67104296, 67104297, 67104298, 67104308, 67104314, 67104315, 67104323, 67104329, 67104341, 67104351, 67104364, 67104375, 67104390, 67201090, 67201627, 67202616, 67202697, 67202721, 67203099, 67262438, 67262504, 67300667, 67537244, 67537596, 68729332, 76714002, 90974101.

PubChem CIDs for Crizotinib patented analogs:

11575401, 11576617, 11597571, 11612136, 11625675, 11647759, 11647760, 11654090, 11656580, 11662380, 11667754, 117071753, 21110756, 21110757, 44256053, 44256477, 54579455, 56671814, 56671943, 66548953, 71621328, 71664254, 71664255, 72199381, 73386634, 76322034.
